# Neuron-autonomous transcriptome changes upon ischemia/reperfusion injury

**DOI:** 10.1038/s41598-017-05342-9

**Published:** 2017-07-19

**Authors:** Jinlong Shi, Xia Chen, Haiying Li, Youjia Wu, Shouyan Wang, Wei Shi, Jian Chen, Yaohui Ni

**Affiliations:** 1 Jiangsu Clinical Medicine Center of Tissue Engineering and Nerve Injury Repair and Department of Neurosurgery, Affiliated Hospital of Nantong University, 20 Xisi Road, Nantong, 226001 Jiangsu China; 2 Basic Medical Research Center, Medical School, Nantong University, 19 Qixiu Road, Nantong, 226001 Jiangsu China; 3 Department of Pediatrics, Affiliated Hospital of Nantong University, 20 Xisi Road, Nantong, 226001 Jiangsu China; 4 Jiangsu Clinical Medicine Center of Tissue Engineering and Nerve Injury Repair and Department of Neurology, Affiliated Hospital of Nantong University, 20 Xisi Road, Nantong, 226001 Jiangsu China

## Abstract

Ischemic stroke and the following reperfusion, an acute therapeutic intervention, can cause irreversible brain damages. However, the underlying pathological mechanisms are still under investigation. To obtain a comprehensive, real-time view of the cell-autonomous mechanisms involved in ischemic stroke and reperfusion, we applied the next-generation sequencing (NGS) technology to characterize the temporal changes in gene expression profiles using primarily cultured hippocampal neurons under an oxygen-glucose deprivation/reperfusion (OGD/R) condition. We first identified the differentially expressed genes (DEGs) between normal cultured neurons, neurons with OGD, and neurons with OGD followed by reperfusion for 6 h, 12 h, and 18 h, respectively. We then performed bioinformatics analyses, including gene ontological (GO) and pathway analysis and co-expression network analysis to screen for novel key pathways and genes involved in the pathology of OGD/R. After we confirmed the changes of selected key genes in hippocampal cultures with OGD/R, we further validated their expression changes in an *in vivo* ischemic stroke model (MCAO). Finally, we demonstrated that prevention of the up-regulation of a key gene (Itga5) associated with OGD/R promoted hippocampal neuronal survival. Our research thereby provided novel insights into the molecular mechanisms in ischemic stroke pathophysiology and potential targets for therapeutic intervention after ischemic stroke.

## Introduction

Ischemic stroke accounts for the vast majority of all strokes, which are the leading cause of disability and the second leading one of death in developed countries^1, 2^. The major outcome of the ischemic stroke is the insufficient blood flow to the brain, which fails to meet its high metabolic demand^3^. Restoration of blood flow by reperfusion immediately following ischemic stroke is therefore often used as the only acute therapeutic intervention. However, such therapy usually requires a very narrow time window. In addition, reperfusion may exacerbate the injury initially caused by ischemia, producing a so-called “cerebral reperfusion injury”^4, 5^.

No clinically therapeutic protocols are so far proved highly effective to ameliorate the brain damage caused by ischemia and reperfusion. For example, neither lowering entire CNS or local neuronal excitability by barbiturate, calcium or glutamate receptor antagonists nor clearing the radical molecules by tirilazad showed overwhelmingly beneficial effects on clinical treatment of ischemic stroke^6–9^. A possible explanation is that the underlying mechanisms of ischemic stroke are still largely unknown. As a result, more comprehensive analysis of the repertoire of gene expressional changes during ischemia development and progression will significantly promote the development of novel therapies towards ischemic stroke.

In the present study, we explored the gene expression profiles by RNA-Seq analysis in primarily cultured hippocampal neurons of oxygen-glucose deprivation/reperfusion (OGD/R) conditioning, which was widely used to mimic brain ischemia and reperfusion^10^. We then performed gene ontological (GO) and pathway analysis of the differentially expressed genes (DEGs) and established co-expression networks to screen key regulatory genes in primary neurons after OGD/R. The expressional changes of selected key genes at various time points were further validated both after OGD/R in cultured hippocampal neurons and in an *in vivo* ischemic stroke model (MCAO/R). Furthermore, we showed that inhibiting one key gene (Itga5) that was significantly up-regulated at acute phase upon OGD/R successfully prevented neuronal apoptosis and thereby promoted cell viability after OGD. By applying stringent bioinformatics analyses, *in vivo* model validation and gene expression manipulation, our research elaborated previous studies for candidate gene screening of the ischemic stroke and therefore provides novel insights into the multifactorial therapeutic approaches to treat ischemic stroke and reperfusion caused brain damages.

## Materials and Methods

### Cell culture, transfection and OGD/R treatment

All experiments were approved by the Ethics Committee of the Affiliated Hospital of Nantong University and followed the guide for the care and use of laboratory animals and other NIH guidelines. The rat hippocampal cells were isolated as described previously^11^. In brief, after E18-E19 Sprague-Dawley (SD) rat embryos (obtained from the experimental animal center of Nantong University) were sacrificed by cervical dislocation under anesthesia, brains were quickly removed, and the hippocampi were harvested on a cold stage. Hippocampal tissues were mechanically and enzymatically dissociated to get single cell suspensions. Hippocampal cells were then plated onto poly-lysine-coated plates at the density of 1 × 10^6^ per ml in DMEM supplemented with 10% FBS (Invitrogen, GrandIsland, NY) and incubated for 4 h at 37 °C in a humidified atmosphere of 95% air and 5% CO_2_. After cells were attached, the medium was changed to Primary Neuron Basal Medium with 2% B27 supplement (Invitrogen, GrandIsland, NY), followed by incubation for 7–8 days with half of the medium being changed every 3 days.

To transfect primarily cultured neurons, control siRNA (ACGUGACACGUUCGGAGAA, 200 nM), siRNA-1 (CGGCACAGCCATGGAAAAA-Cy3, 200 nM) & siRNA-2 (CTCGGCTTCTTCAAACGCT-Cy3, 200 nM) against Itga5 were mixed with Lipofectamine® RNAiMAX Reagent (1:1 ratio, Thermo Fisher Scientific, 13778030) in opti-MEM and added directly into the DMEM culture medium. Culture medium was changed at 16 h after transfection. To optimize the transfection efficiency, we mixed siRNAs of multiple concentrations with Lipofectamine® RNAiMAX and used the immunofluorescence as an indicator (Supplementary Fig. 1). Forty-eight hour after transfection, cultures were treated for OGD-R injury.

Before inducing OGD-R injury, cultured hippocampal neurons were rinsed twice with PBS and maintained in glucose-free DMEM. Cells were then placed into a hypoxic incubator (Don Whitley Scientific, England) with 1% O_2_, 5% CO_2_, and 94% N_2_ for 45 min at 37 °C to mimic OGD injury^12, 13^, (named OGD 45 min/R 0 h). Control cells (OGD 0 min/R 0 h) were harvested after incubating with DMEM with glucose in a humidified incubator with 5% CO_2_ at 37 °C for the same times. After 45 min hypoxia challenge, some hippocampal cells were transferred to Primary Neuron Basal Medium with 2% B27 supplement and returned back into normoxic conditions for 6 h (OGD 45 min/R 6 h), 12 h (OGD 45 min/R 12 h) or 18 h (OGD 45 min/R 18 h) before harvest to mimic reperfusion caused injury.

### RNA extraction and RNA-Seq analysis

Total RNA was extracted using Trizol reagent (Invitrogen, Carlsbad, CA), following the manufacturer’s instruction. RNA by the Agilent 2200 Bioanalyzer. Samples were processed using the Illumina mRNA-Seq Sample Preparation Kit (containing 1 Box, part # 1004824 and 1 Bag, part # 1004825) according to the manufacturer’s protocol. Briefly, polyA containing mRNA was enriched by oligo-dT magnetic beads from 5 μg total RNA and fragmented into small pieces using divalent cations at 94 °C. The first-strand cDNA was synthesized by reverse transcriptase and random primers. RNA was then removed by RNaseH and the second-strand cDNA was synthesized by DNA polymerase I. The cDNA fragments then underwent an end repair process, the addition of a single ‘A’ base, and the ligation of the adapters. The products were then purified and PCR amplified to create final cDNA libraries. RNA-seq libraries were 100 bp, paired-end sequenced on an Illumina HiSeq 2000. Sequencing reads after removing polymers, primer adaptors, and ribosomal RNAs were aligned to rat genome with SOAPaligner/SOAP2^14^. The alignment data is utilized to calculate distribution of reads on reference genes and perform coverage analysis. The expression level for each gene was measured by the reads per kilo-base per million (RPKM)^15, 16^ after quality controls. Genes were considered significantly differentially expressed if they exhibited at least a two-fold difference in expression with a false discovery rate (FDR) less than 0.001. The original CEL format data were deposited at bioproject at NCBI, with link as: https://www.ncbi.nlm.nih.gov/bioproject/?term = PRJNA376061. The complete differentially expressed genes (DEG) list for each time points was listed in Supplementary Table 1.

### Gene Ontology (GO) analysis

All differentially expressed genes (DEGs) were mapped to GO terms in the database (http://www.geneontology.org/). Hypergeometric test was applied to find significantly enriched GO terms in the input list of DEGs, based on ‘GO::TermFinder’ (http://smd.stanford.edu/help/GO-TermFinder/GO_TermFinder_help.shtml). Bonferroni Correction was applied to adjust the p-value^17^. FDR adjusted p-value ≤ 0.05 was used as a threshold and. GO terms fulfilling this condition were defined as significantly enriched.

### Pathway analysis

Pathway analysis was used to identify significant pathways involving differential gene expression, according to Kyoto Encyclopedia of Genes and Genomics (KEGG)^18^. Pathways with FDR adjusted p-value ≤ 0.05 were defined as significantly enriched. Cytoscape were used to generate graphical representations of pathways^19^.

### Gene-act-network and gene co-expression analyses

The DEGs in the significantly enriched GO terms and pathways were selected to build gene co-expression networks (gene-act-net). For each pair of genes, we calculated the Pearson’s correlation and chose the significantly correlated pairs to construct the network^20^, according to their normalized signal intensities. In network analysis, degree centrality is the simplest and most important measure of the centrality of a gene within a network, determining its relative importance. Degree centrality is defined as the number of links one node has with another^21^. Additionally, to study the properties of the constructed gene networks, k-cores in graph theory were introduced as a method of simplifying graph topology analysis. The k-core of a network is a sub-network in which all nodes are connected to at least k other genes in the sub-network. During our analysis of different networks, core regulatory factors were determined by the degree of differences between the samples obtained from the two groups^22^.

### Quantitative real-time reverse transcription polymerase chain reaction (qPCR)

Reverse transcription was performed with the High-Capacity cDNA Reverse Transcription Kits (Applied Biosystems, Foster City, CA, USA), according to the manufacturer’s instruction. qPCR was performed on an ABI 7500 thermocycler (Applied Biosystems, Foster City, CA, USA) by using SYBR Green Real-Time PCR Master Mix (Toyobo, Japan). GAPDH was used for normalization. All quantitative PCR reactions were performed in biological triplicates. Primer sequences were listed in Supplemental Table 2.

### Cell Viability and TUNEL assay

The cell viability of hippocampal neuron cells was evaluated using thereduction 3-(4,5-Dimethyl-2-thiazolyl)-2,5-diphenyl-2H-tetrazolium bromide (MTT) assay as described previously. In brief, the 96-well culture plates were seeded in hippocampal neurons with a density of 1 × 10^6^ cells/ml for 7 days. After pretreatment with control, siRNA-1 & 2 against Itga5 for 48 h, all groups were exposed to OGD45min/R18h except the normal control (OGD0min/0 h). Each well was then added with 10 ul of MTT solution (5 mg/ml), incubated for 4 h, and followed by adding the 20% SDS solution (100 ul) for 20 h. Spectrophotometry was used to measure the absorbance at 570 nm by Tecan M200 Microelisa reader (Tecan company, Austria). TUNEL assay was performed according to manufacture’s instructions. Briefly, cultured cells were washed (1 × PBS), fixed (2% paraformaldehyde, 1 × PBS), and permeated (0.1% Triton X-100), and incubated with TUNEL reaction mixture (*in situ* cell death detection kit, Roche). Results were analyzed by fluorescence microscope.

### Middle cerebral artery occlusion (MCAO)/reperfusion (R) model

Healthy male Sprague-Dawley (SD) rats (n = 20) with body weights of 250 ± 30 g were provided by the experimental animal Center of Nantong University (Jiangsu Provience, China). Rats were randomly divided into five groups: the sham operation group (n = 4), the MCAO2h/R0h group (n = 4), the MCAO2h/R6h group (n = 4), the MCAO2h/R12h group (n = 4) and MCAO2h/R24h group (n = 4).

The procedure of the MCAO/R was described elsewhere. Briefly, before the surgery, rats were fasted for 12 h, water deprived for 5 h and maintained spontaneous activities. With the 10% chloral hydrate (0.3 ml/100 g, i.p) anesthetized, rats were fixed on the surgical splint with supine position and the common carotid artery (CCA), the internal carotid artery (ICA) and the external carotid artery (ECA) were exposed following the median incision on the neck. The CCA and ECA were then tied up with a surgical suture respectively, and the ICA was clamped by an artery clip. In next step, a small incision was made in the CCA, and a single strand of nylon thread was gently inserted into the ICA (about 18.5 ± 0.5 mm) via the CCA until feeling slight resistance, which suggested that the nylon thread completely blocked the origin of the middle cerebral artery in the circle of Willis. After that, maintaining the nylon thread fastened by a surgical suture in its position for 2 hours, the thread was removed carefully to recover the blood flow and then achieved the reperfusion. During surgery, all rats body temperature should pay attention to maintaining at 37 ± 0.5 °C.

### Western blot

With the different time reperfusion, the rats were sacrificed and the affected cortical and subcortical areas were quickly separated from the brain on dry ice, weighed, and fully homogenized. Proteins and mRNAs were extracted, respectively. For proteins, the concentration was measured by BCA protein assay kit (Beyotime Institute of Biotechnology, Shanghai, China) and then dissolved in loading buffer with equal amount (25 ug) and run in the SDS-PAGE gel. The gel was transferred onto the polyvinylidene difluoride filer (PVDF) membrane. Blots were blocked with 5% non-fat powdered milk in tris-buffered saline for 1 h, and incubated with anti-ErbB4 polyclonal antibody (1: 1000 Santa Cruz, sc-283), anti-Itga5 (1: 1000 Santa Cruz, sc-10729) and anti-GAPDH (1: 1000 Santa Cruz Biotechnology Inc) overnight at 4 °C, followed by incubating with secondary antibodies [goat anti-rabbit IgG-HRP (1: 5000), Santa Cruz Biotechnology Inc)], HRP-conjugated goat anti-rat IgG (1: 3000 Biyun days Biotechnology Research Institute) for 2 h at room temperature. The analysis of western blot was made in a chemiluminescence imaging system (BIO-RAD, USA). The GAPDH expression was used as a loading control.

### Statistical analysis

For all figures, error bars figures represent mean ± SEM, the number (n) of samples employed is indicated in legends. Student’s t test, One-way ANOVA with Bonferroni correction for multiple comparisons (all were shown in figure legends) were performed to determine the significance difference between different groups. For all statistics, **p < 0.01, *p < 0.05, n.s., no statistical significance.

## Results

### Profiling gene expression in primarily cultured hippocampal neurons with oxygen-glucose deprivation (OGD) and reperfusion

To compare the transcriptome differences between control, OGD, and OGD with reperfusion, we isolated RNA from primary cultured hippocampal neurons with control treatment (OGD 0 min/R 0 h), oxygen-glucose deprivation for 45 minutes (OGD 45 min/R 0 h), or oxygen-glucose deprivation for 45 minutes followed with 6, 12, or 18 hours of reperfusion (OGD 45 min/R 6 h, OGD 45 min/R 12 h, or OGD 45 min/R 18 h, respectively). More than 58 million high-quality, mappable reads were obtained in each sample, indicating the sufficient depth. After quality control filtering, reads were mapped to the rat genome using SOAPaligner/SOAP2 (the analyses pipeline was summarized in Supplemental Fig. 2). At least 83.08% of the sequences were well aligned to the genome, with over 67.92% localized in mRNA regions (Table 1). Additionally, reads of all samples were evenly distributed on reference genes, indicating high quality of sample processing and sequencing.

**Table 1:** Sequencing statistics of individual comparisons.

	Items	OGD0min/R0h		OGD45 min/R0h		OGD45min//R6h		OGD45min/R12h		OGD45min/R18h	
		number	percentage	number	percentage	number	percentage	number	percentage	number	percentage
**Map to Gene**	**Total Reads**	59128068	100.00%	59076626	100.00%	59223558	100.00%	59304994	100.00%	58426558	100.00%
	**Total BasePairs**	5321526120	100.00%	5316896340	100.00%	5330120220	100.00%	5337449460	100.00%	5258390220	100.00%
	**Total Mapped Reads**	40301339	68.16%	42052324	71.18%	40641556	68.62%	40700247	68.63%	39681482	67.92%
	**perfect match**	32075475	54.25%	33670782	57.00%	32907931	55.57%	33067872	55.76%	32147019	55.02%
	**<=5bp mismatch**	8225864	13.91%	8381542	14.19%	7733625	13.06%	7632375	12.87%	7534463	12.90%
	**unique match**	38914017	65.81%	40601334	68.73%	38987738	65.83%	38888128	65.57%	37667148	64.47%
	**multi-position match**	1387322	2.35%	1450990	2.46%	1653818	2.79%	1812119	3.06%	2014334	3.45%
	**Total Unmapped Reads**	18826729	31.84%	17024302	28.82%	18582002	31.38%	18604747	31.37%	18745076	32.08%
**Map to Genome**	**Total Reads**	59128068	100.00%	59076626	100.00%	59223558	100.00%	59304994	100.00%	58426558	100.00%
	**Total BasePairs**	5321526120	100.00%	5316896340	100.00%	5330120220	100.00%	5337449460	100.00%	5258390220	100.00%
	**Total Mapped Reads**	49412149	83.57%	49078920	83.08%	49453425	83.50%	49529030	83.52%	48750289	83.44%
	**perfect match**	37009834	62.59%	36843692	62.37%	37577522	63.45%	37846334	63.82%	37217026	63.70%
	**<=5bp mismatch**	12402315	20.98%	12235228	20.71%	11875903	20.05%	11682696	19.70%	11533263	19.74%
	**unique match**	45681539	77.26%	45079783	76.31%	45197916	76.32%	45023931	75.92%	44060025	75.41%
	**multi-position match**	3730610	6.31%	3999137	6.77%	4255509	7.19%	4505099	7.60%	4690264	8.03%
	**Total Unmapped Reads**	18826729	31.84%	9997706	0.1692	9770133	16.50%	9775964	16.48%	9676269	16.56%

**Figure 1:**
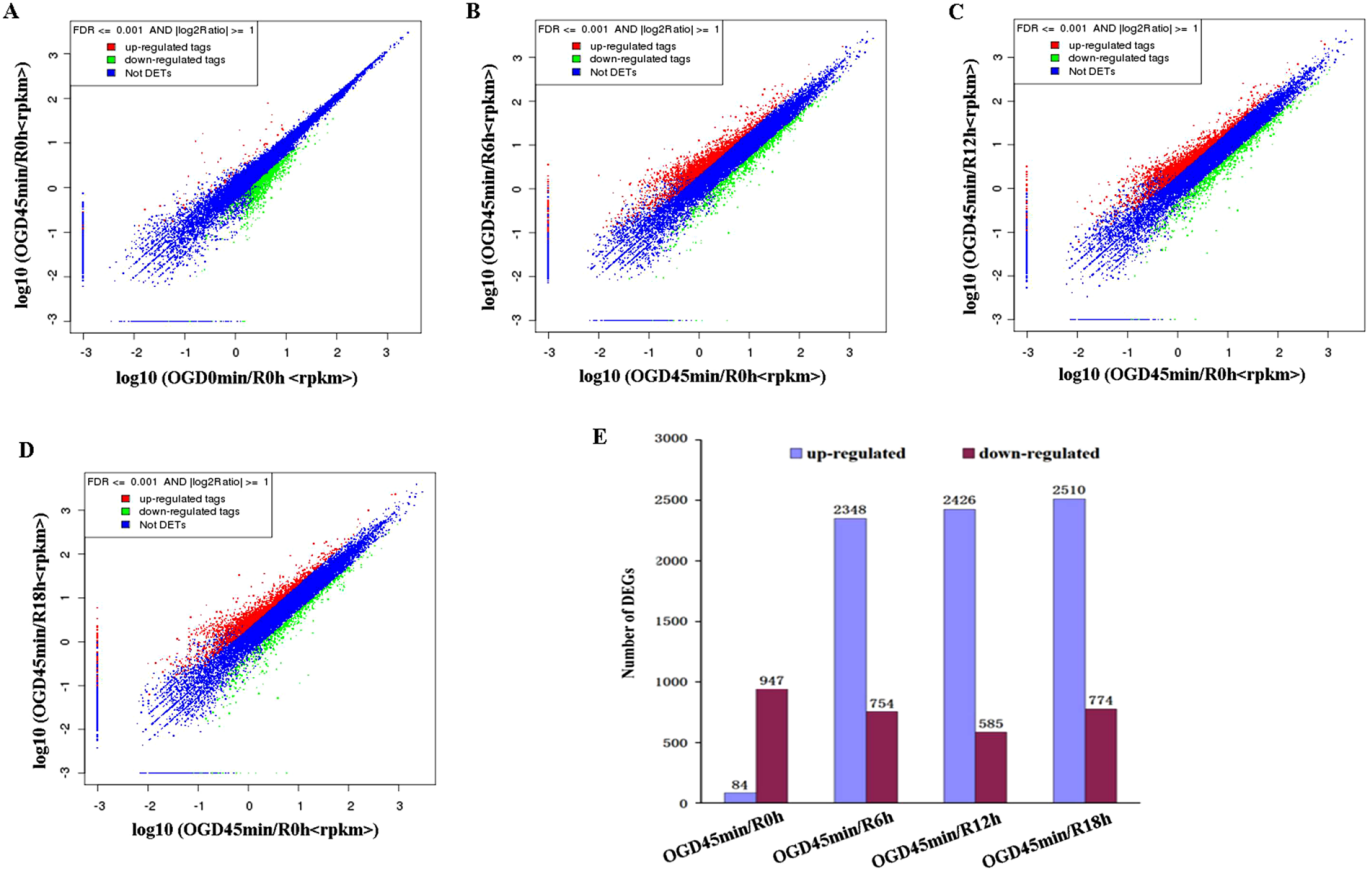
Comparisons of gene expression profiles of primarily cultured hippocampal neurons upon oxygen glucose deprivation (OGD), and OGD with reperfusion. (**A–D**) The overview of gene expression profiles of OGD 45 min/R 0 h vs OGD 0 min/R 0 h (**A**); OGD 45 min/R 6 h vs OGD 45 min/R 0 h (**B**); OGD 45 min/R 12 h vs OGD 45 min/R 0 h (**C**); OGD 45 min/R 18 h vs OGD 45 min/R 0 h (**D**). (**E**) Bar graph demonstrating the quantity of positively and negatively expressed transcripts upon OGD and OGD with reperfusion (FDR ≤ 0.001 and a fold change > 2).

After calculating the gene expression levels of each sample by using RPKM method, we compared gene expression profiles between individual conditions (OGD 45 min/R 0 h vs OGD 0 min/R 0 h, OGD 45 min/R 6 h vs OGD 45 min/R 0 h, OGD 45 min/R 12 h vs OGD 45 min/R 0 h, and OGD 45 min/R 18 h vs OGD 45 min/R 0 h) (Fig. 1A–D). We used FDRuseGD4 and a fold change > 2 as the thresholds for the significant difference. A total of 1031 transcripts were differentially expressed between OGD 45 min/R 0 h and OGD 0 min/R 0 h. Of these genes, 947 were up-regulated and 84 were down-regulated. When compared to OGD only, a total of 3102, 3011 and 3284 differentially expressed transcripts were identified at different time points after reperfusion (Fig. 1E).

### Functional enrichment analysis of DEGs

**Figure 2:**
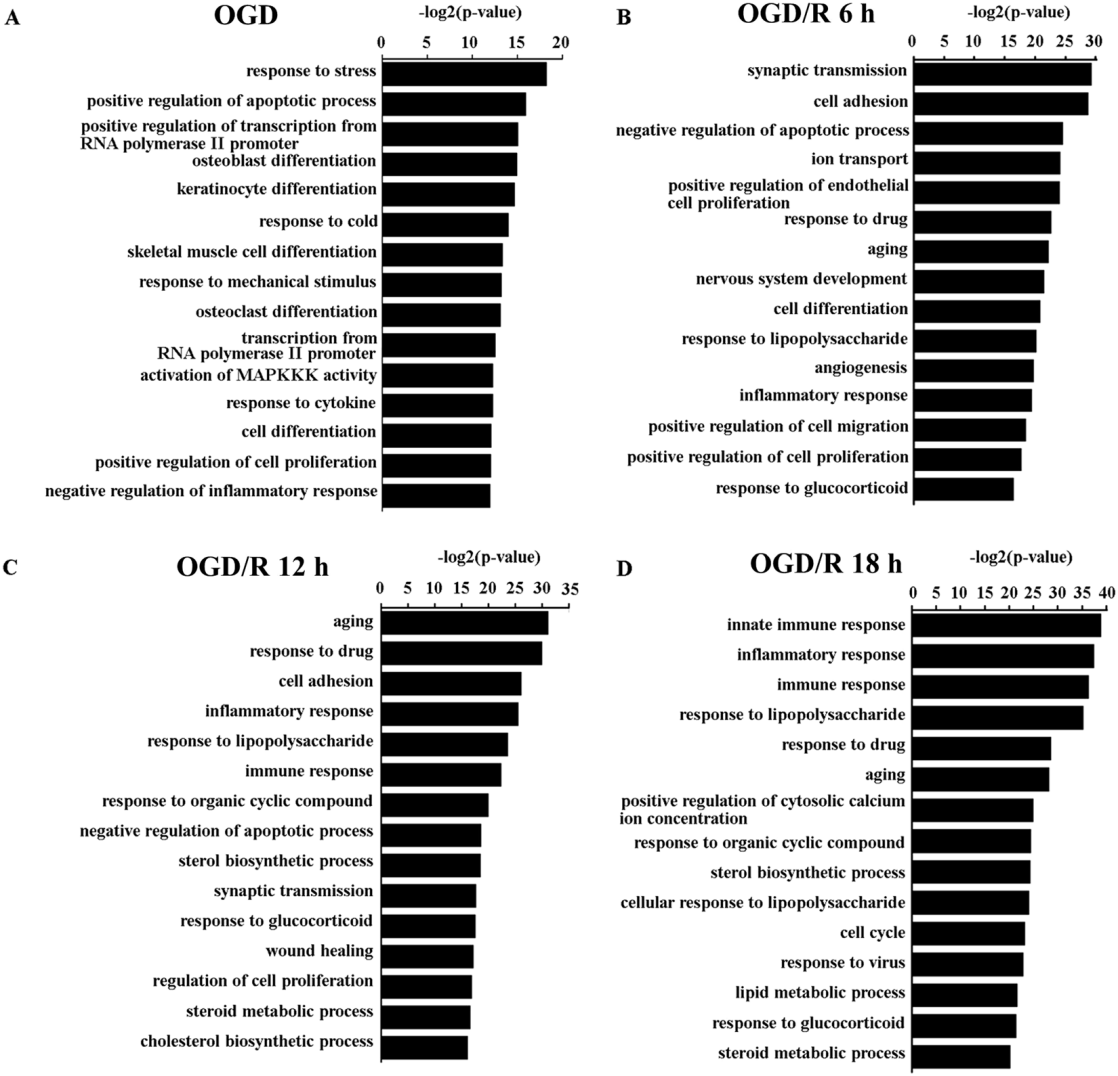
Gene ontology (GO) enrichment analysis of genes differentially expressed in primarily cultured neurons upon oxygen glucose deprivation (OGD), and OGD with reperfusion. (**A**–**D**) GO terms with most significant P-value were shown for OGD 45 min/R 0 h vs OGD 0 min/R 0 h (**A**); OGD 45 min/R 6 h vs OGD 45 min/R 0 h (**B**); OGD 45 min/R 12 h vs OGD 45 min/R 0 h (**C**); and OGD 45 min/R 18 h vs OGD 45 min/R 0 h (**D**).

**Figure 3:**
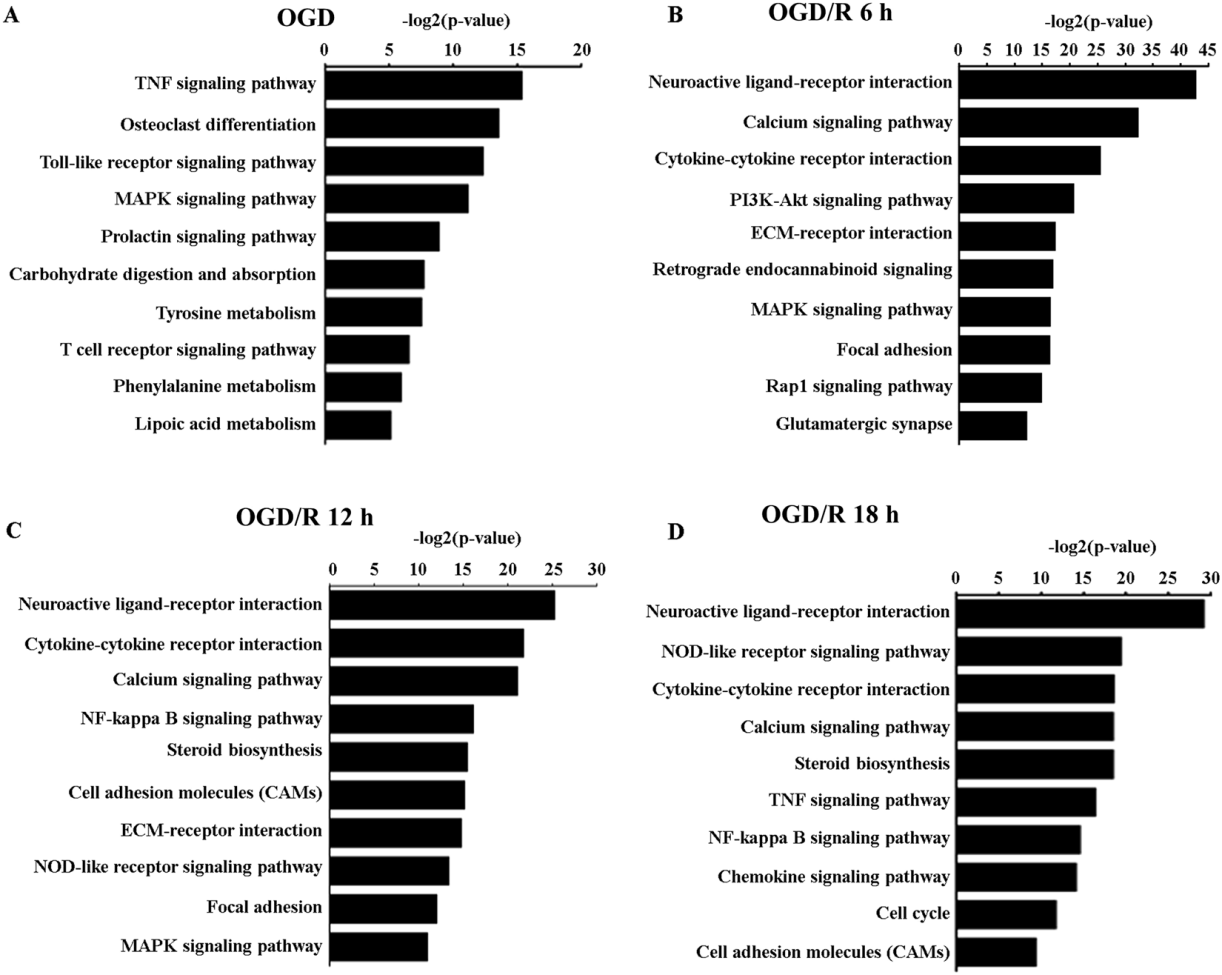
Pathway enrichment analysis of genes differentially expressed in primarily cultured neurons upon oxygen glucose deprivation (OGD) and OGD with reperfusion. (**A–D**) Regulated pathway terms with most significant P-value were shown in for OGD 45 min/R 0 h vs OGD 0 min/R 0 h (**A**); OGD 45 min/R 6 h vs OGD 45 min/R 0 h (**B**); OGD 45 min/R 12 h vs OGD 45 min/R 0 h (**C**); and OGD 45 min/R 18 h vs OGD 45 min/R 0 h (**D**).

To identify potential biological processes associated with OGD and reperfusion injury, we performed bioinformatics analyses on annotated genes (DEG) within those differentially expressed transcripts (Supplementary Table 1). GO analysis showed that OGD induced strong stress and apoptosis responses, along with a global transcription elevation (Fig. 2A). Pathway-based analysis (KEGG) further highlighted the TNF signaling pathway and the Toll-like receptor signaling pathway, both of which were involved in inflammatory response upon stimulation^23, 24^ (Fig. 3A, Supplemental Fig. 3).

At acute phase of reperfusion, the most enriched biological processes of DEGs were related to synaptic transmission and cell adhesion (Fig. 2B). In contrast, the DEGs were mainly enriched in the biological processes closely related to metabolism regulation such as sterol biosynthetic process, lipid metabolic process, at a relatively late phase of reperfusion (Fig. 2C,D).

Pathways relating to cell survival (such as calcium signaling pathway, neuroactive ligand-receptor interaction) and inflammation response (NOD-like receptor signaling pathway, TNF signaling pathway, NF-kappa B signaling pathway) were continuously activated at different phases of reperfusion (Fig. 3B–D). In contrast, the activation of MAPK pathway, which was significantly enriched at the beginning of OGD and reperfusion gradually faded at late phase of reperfusion (Fig. 3A–D, Supplemental Figs 3–6).

### Identification of key genes from co-expression network

To explore the relationships among those DEGs, we established the act networks of DEGs (Supplemental Figs 7–10). The results highlighted several key gene networks that were general altered in neurons after OGD/R. For example, multiple integrin proteins and their interacted proteins were up-regulated at all three time points after reperfusion. These results, consistent with previous reports^25, 26^, suggested that integrin cell surface receptors play important roles in stroke pathophysiology. We then selected the potential key genes involved in OGD/R injury for validation using three criteria. First, core regulatory DEGs involved in the window of ischemia and reperfusion for different duration were determined by the degree of differences (log2 Ratio > 1.5); Second, the core regulatory DEGs involved in corresponding enriched pathway were selected; Third, the DEGs related to the known pathologic mechanisms (for example, cytoplasm calcium overload and excess oxygen radical production^27^) of OGD were selected. Based on above criteria, we chose 9 key DEGs from OGD 45 min neurons, 12 key DEGs from OGD 45 min/R 6 h, 12 key DEGs from OGD 45 min/R 12 h neurons, and 9 key DEGs from OGD 45 min/R 18 h neurons (Supplemental Tables 3–6).

**Figure 4:**
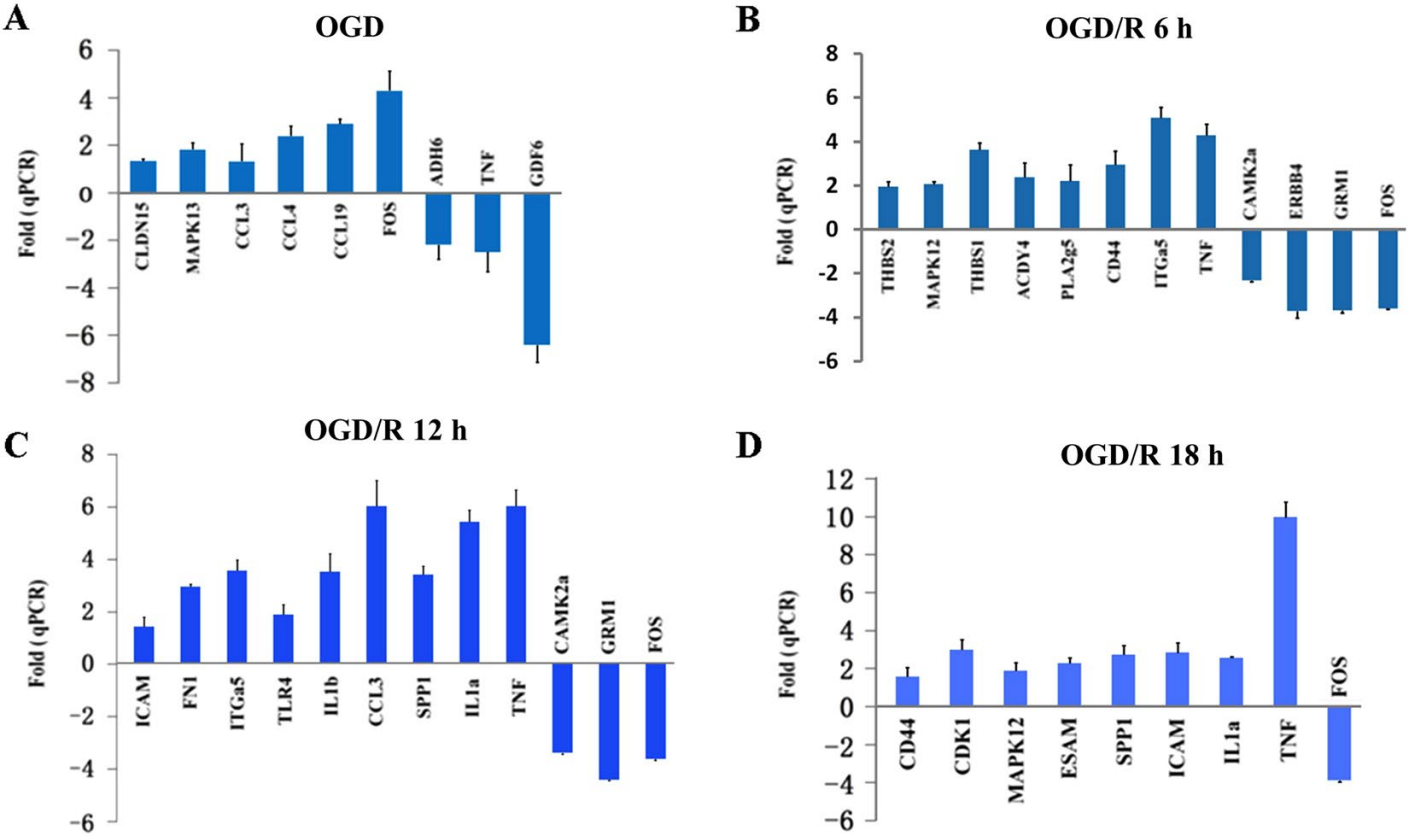
Validation of key regulatory genes involved in oxygen glucose deprivation (OGD) pathophysiology. **(A–D**) The selected differentially expressed genes (DEGs) for OGD 45 min/R 0 h vs OGD 0 min/R 0 h (**A**); OGD 45 min/R 6 h vs OGD 45 min/R 0 h (**B**); OGD 45 min/R 12 h vs OGD 45 min/R 0 h (**C**); and OGD 45 min/R 18 h vs OGD 45 min/R 0 h (**D**) were validated by qPCR. For each time point, neurons were collected and pooled from hippocampal cells isolated from six embryos. Triplicates (from pooled RNAs) were then performed.

We validated the expressional changes of these key DEGs by quantitative PCR (Fig. 4A–D). Overall, the quantitative RT-PCR data and the RNA-seq results showed high coefficient, indicating the RNA-seq results were highly reliable (Supplementary Fig. 11).

### Validation of expression changes of selected key genes in an *in vivo* ischemic stroke model

**Figure 5:**
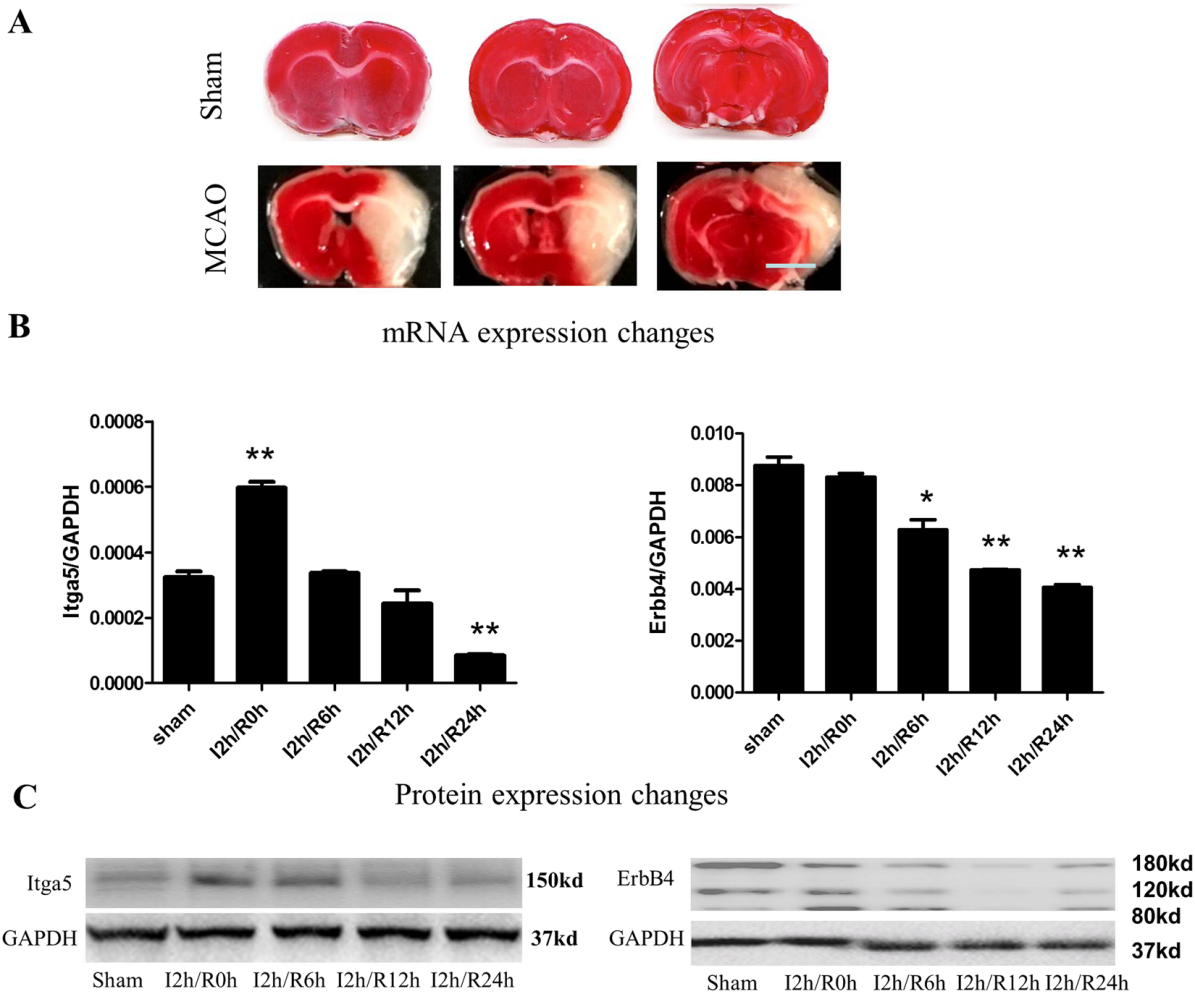
Validation of identified key gene expression changes after ischemic stroke (MCAO) (**A**) Representative images of TTC staining showing the infarct area in sham control or MACO treated animals. Scale bar: 1 mm. (**B**,**C**) mRNA (qRT-PCR) and protein (western blotting) expression changes of two identified key genes (Itga5 and ErbB4) at various time points after ischemic stroke (MCAO). **p < 0.01, One-way ANOVA followed by Bonferroni post hoc correction (against sham control), n = 3 for each time points (samples were pooled from 4 animals for individual time points) for sham, 12 h/R 0 h, 12 h/R 6 h, 12 h/R 12 h, and 12 h/R 24 h groups.

Although powerful to examine the cell autonomous effects, a key issue for *in vitro* assays is how the results correlate with the *in vivo* scenarios. To validate our finding *in vivo*, we applied the middle cerebral artery occlusion (MCAO) stroke model. In this model, the middle cerebral artery was temporarily blocked and followed by reperfusion. Our results indicated that 2 h occlusion of this artery induced significant ischemia, with a large infarction area including the ipsilateral motor, somatosensory cortex and subcortical striatum (Fig. 5A). To compare the gene expressional change between the primarily cultured neurons after OGD and neurons in ischemic stroke model *in vivo*, we selected two key genes [integrin-alpha-5 (Itga5); Receptor tyrosine-protein kinase (ErbB-4), Fig. 4] that were up-regulated and down-regulated after OGD, respectively. The quantitative-RT-PCR results indicated that the mRNA levels of Itga5 and ErbB4 showed a temporally increase and gradual decrease following the MCAO (Fig. 5B). Consistently, the Itga5 protein expression was immediately up-regulated and last for 6 h after MCAO, whereas the ErbB4 protein started to decrease at 6 h after MCAO (Fig. 5C). These data showed that the expression changes of selected key genes in cultured neurons after OGD were well recapitulated in an *in vivo* ischemic stroke model, further demonstrating that our screening with an *in vitro* OGD condition provided valuable insights into the molecular mechanisms of the pathophysiology of the ischemic stroke.

### Down regulation of Itga5, an identified key gene, inhibits neuronal apoptosis after OGD

**Figure 6:**
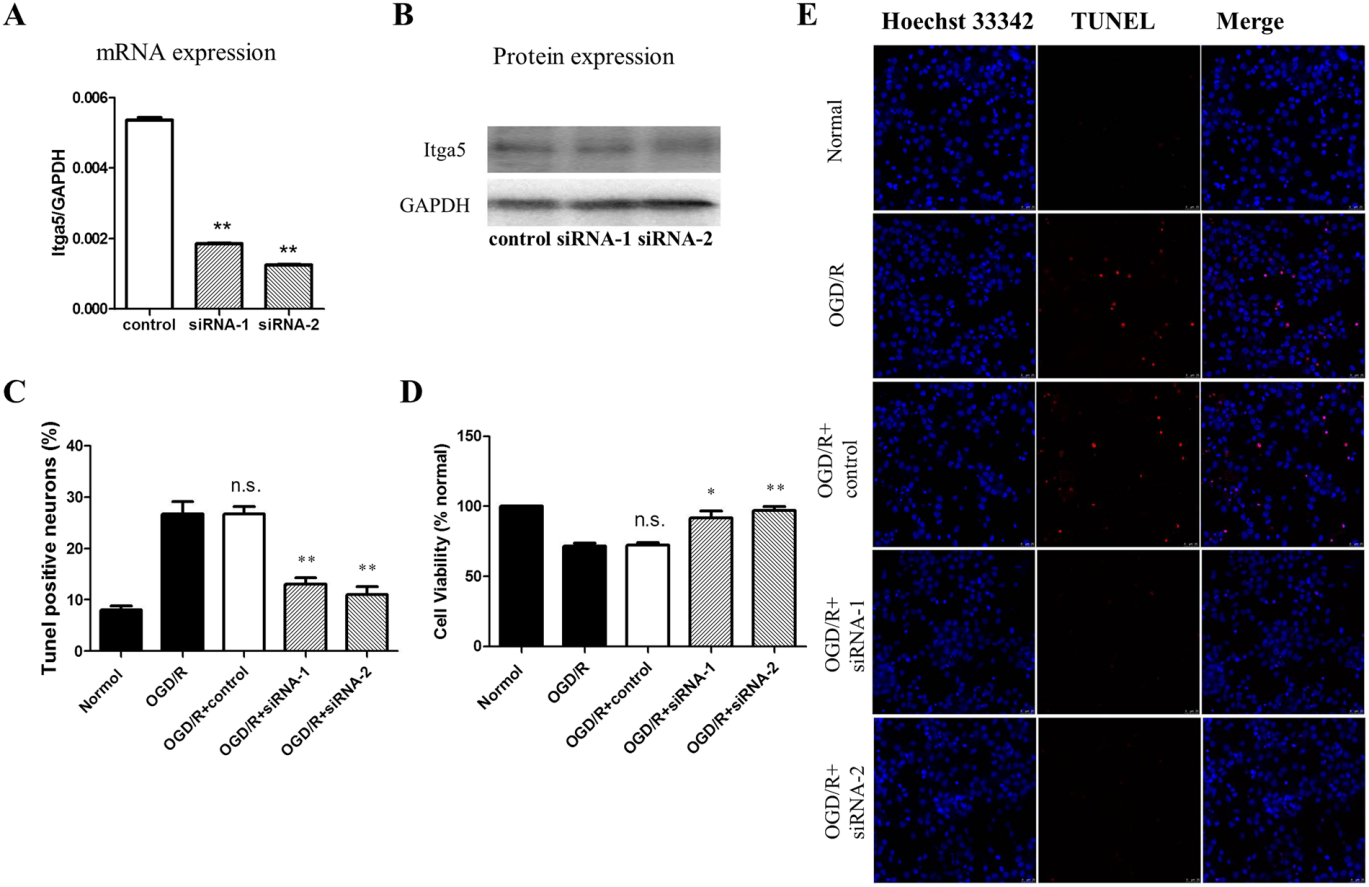
Itga5 downregulation promoted neuronal survival after OGD/R. (**A**,**B**) mRNA quantification (**A**) and Western blotting (**B**) of Itga5 expression under control, siRNA1 (against Itga5) treatment. **p < 0.01, One-way ANOVA followed by Bonferroni post hoc correction, n = 3 for each condition (RNAs and proteins were pooled from 6 cultures for each condition). (**C**,**D**) Percentage of neuronal apoptosis (TUNEL staining, (**C**) and cell viability (**D**) in normal, OGD/R, OGD/R with control, siRNA1 & 2 (against Itga5) conditions. **p < 0.01, One-way ANOVA followed by Bonferroni post hoc correction (against OGD/R condition), n = 6 for each condition. (**E**) Representative images of the staining of Hoechst 33342, TUNEL, and their merge in normal, OGD/R, OGD/R with control, siRNA1 & 2 (against Itga5) treated.

To study the pathophysiological roles of the gene profiling changes post OGD, we targeted one key gene (Itga5) that was sharply up-regulated at the acute phase during the reperfusion after OGD (Fig. 4B,C, and Supplementary Tables). By adding siRNAs against Itga5, we successfully knocked down its expression in primarily cultured hippocampal neurons (Fig. 6A,B). We then applied the OGD/R to those cultures. The TUNEL assay revealed that the neuronal apoptosis was significantly inhibited when the expression of Itga5 was reduced (Fig. 6C,E). Consistently, the cell viability increased in siRNA-1 and siRNA-2 added groups (Fig. 6D). These results indicated that the transient, cell autonomous Itga5 up-regulation might play a deleterious role in ischemic/reperfusion conditions, since blocking such up-regulation promoted neuronal survival.

## Discussion

Ischemic stroke commonly causes severe disability in surviving victims^1, 2^. Understanding the molecular pathology of stroke injuries, including the injuries caused by ischemia and the following reperfusion, may lead to novel therapies and rehabilitation training after stroke. A majority of current studies mainly use rodent injury models to mimic ischemic stroke^10, 27^. Although more physiological when compared to *in vitro* models, the molecular and cellular changes observed using *in vivo* models could be caused by numerous factors ranging from central nervous system, surrounding vascular, and immune systems, which often made the results difficult to be interpreted. In contrast, *in vitro* assays establish appropriate conditions for the investigation of cell autonomous responses upon ischemia and reperfusion. In this study, by applying OGD/R on primarily cultured rat hippocampal neurons to mimic ischemia and reperfusion condition, we investigated the temporal, cell autonomous transcriptome changes after OGD/R. By validating key gene expressional changes in an *in vivo* stroke model, we further demonstrated that our data provide useful information in studying the underlying mechanisms of the ischemic stroke.

**Figure 7:**
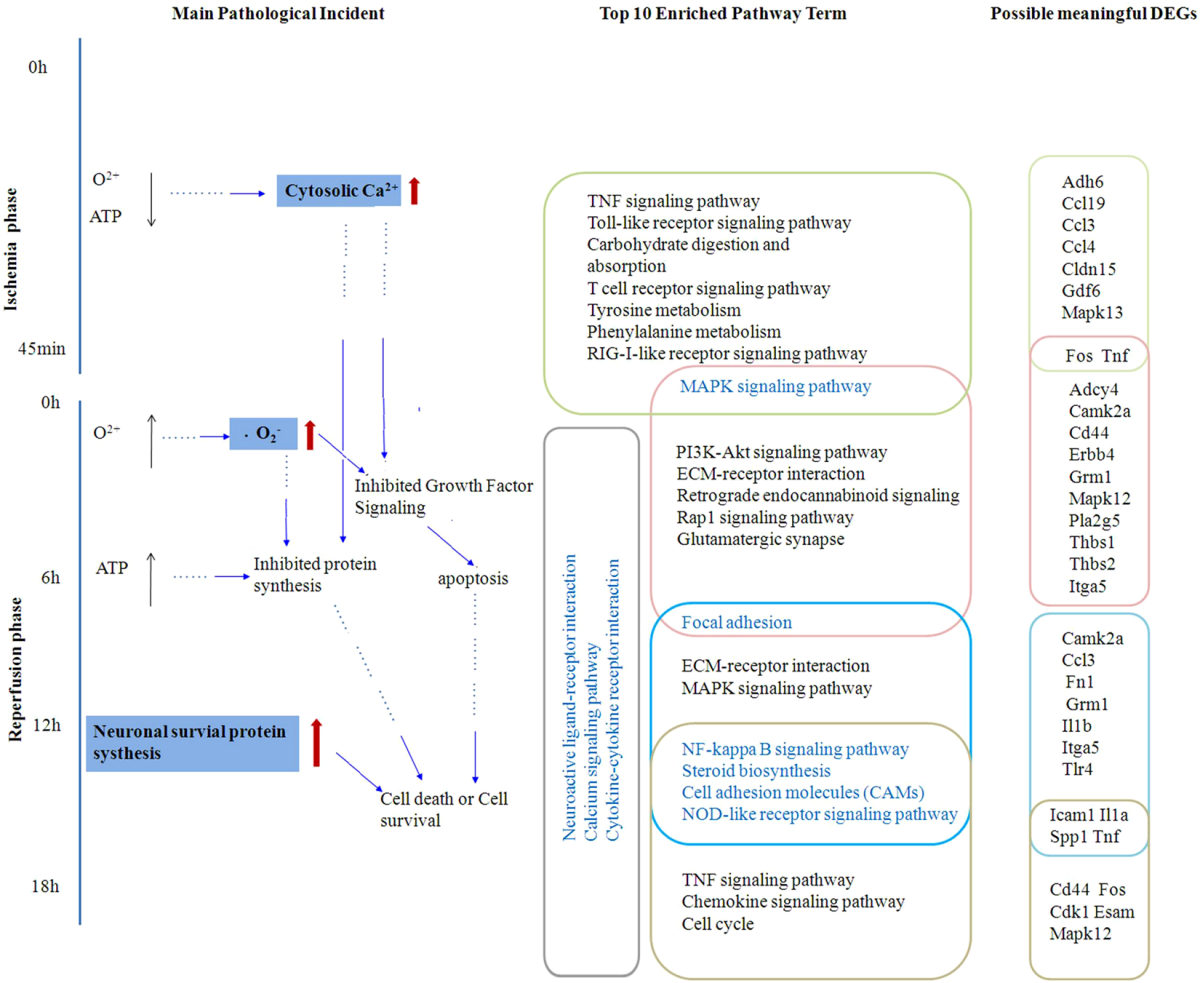
The transition of gene expression patterns in primarily cultured hippocampal neurons after oxygen glucose deprivation (OGD) with reperfusion. Left column, A schematic diagram illustrating major molecular and cellular events upon ischemia and reperfusion. Middle column, Most enriched pathways activated at different time points of ischemia and reperfusion phases. Right column: Validated genes associated with different signaling pathways at different time points of ischemia and reperfusion phases.

Brain ischemia and reperfusion engage multiple pathophysiological steps. By measuring the temporal alteration of transcriptome at different time points of reperfusion, we observed continuous shifts of gene expression profiles. As summarized in Fig. 7, multiple pathways were activated at different stages of reperfusion, with overlaps between adjacent time points. Among them, the calcium signal pathway was continuously activated during ischemia and the following reperfusion. It is known that ischemia causes a rapid loss of high-energy phosphate compounds, generalized depolarization, and increased neuronal cytosolic Ca^2+^, which leads to the dysfunction of cytosolic proteins and signal chemicals, including μ-calpain, calcineurin, NOS, and free arachidonic acid that will further exacerbating the situation^27–30^. Our results indicated that, to buffer or clear overloaded calcium, the related pathways were activated even long after reperfusion. In contrast, the TNF signal pathway showed biphasic activation. It has been well known that TNF pathway is activated during ischemia and contributes to ischemia caused injury^31^. Here we found that a second wave of the enhancement of TNF pathway appeared at reperfusion for 18 hours, long after its previous activation during the ischemia, probably involved in survival signaling transduction^32^. Such results suggested distinctive mechanisms might be involved for the biphasic activation of TNF signaling pathway during ischemia/reperfusion.

Traditional views supported the idea that the inflammation after OGD is mainly caused by the activation of endothelial cells, astrocytes, and microglia, which secrete pro-inflammatory mediators^33^. However, our results showed that, without the vascular system, the inflammatory responses were still activated immediately after OGD and remained active at different phases of reperfusion. Such results suggested a neuronal, cell autonomous role in the pathological, inflammation process after the ischemic stroke insult.

By performing gene-act-network and gene co-expression analyses, we also identified novel, potential key regulators during ischemia and reperfusion. For example, in both *in vitro* OGD/R and *in vivo* ischemic stroke model (MCAO/R) models, integrin-alpha-5 (Itga5) and receptor tyrosine kinase (ErbB4) were up-regulated or down-regulated after reperfusion, respectively.

Integrins, a group of cell surface trans-membrane glycoprotein receptors, are crucial extracellular matrix components involved in cell survival, proliferation and differentiation^34^. Post global hypoxia or ischemic stroke, the expression of α5β1 integrin is significantly up-regulated in brain endothelial cells^25, 26^. Interestingly, recent findings revealed that conditional knockout of Itga5 in endothelial cells significantly reduced the ischemic infarct areas in a transient MCAO model and thereby promoted functional outcome, probably by enhancing the integrity of the brain blood barrier (BBB) after stroke^35^. Therefore, the up-regulation of Itga5 in endothelia cells plays a deleterious role in stroke pathophysiology. Our study expanded the deleterious role of Itga5 to neurons, since knockdown of Itga5 promoted neuronal survival after OGD. Therefore, a local inhibition of Itga5 of both brain endothelia cells and cortical neurons at acute or sub-acute phase after ischemic stroke might be a promising therapeutic approach for further testing.

As a member of the epidermal growth factor receptor subfamily, ErbB4 has been shown to play key roles in the regulation of neurite outgrowth, axonal guidance and synaptic signaling, and plasticity^36, 37^. The precise role of ErbB4 in brain ischemia remains largely unknown. Interestingly, it has been reported that NRG reduced ROS levels in H_2_O_2_ -treated PC12-ErbB-4 cells^38^. On the other hand, NRG protects against ischemic brain injury through an ErbB4-dependent manner^39^. Our results suggested that ErbB4 itself is related to a burst of excess oxygen radical production during early reperfusion. A future direction is therefore examining the effects of manipulating ErbB4 expression on the pathophysiology of the ischemic stroke.

In conclusion, by performing comprehensive gene expression profiling of *in vitro* cultured hippocampal neurons during ischemia and reperfusion, we revealed continuous transitions of transcriptome and identified potential key pathways and genes in ischemia and reperfusion. Our study therefore advanced current understanding of molecular mechanisms in ischemic stroke, which shed light on the discovery of therapeutic avenues to treat ischemic stroke.

## Electronic supplementary material

Supplementary Information

Supplementary Table 1
