# Supplementary material for: Neuron-autonomous transcriptome changes upon ischemia/reperfusion injury
Source: Sci Rep. 2017 Jul 19;7:5800. doi: 10.1038/s41598-017-05342-9 (PMC5517505; doi:10.1038/s41598-017-05342-9)
Supplement: Supplementary file 1 — Supplementary Information [file 41598_2017_5342_MOESM1_ESM.pdf]

Neuron-autonomous transcriptome changes upon ischemia/reperfusion injury

Jinlong Shi[1*], Xia Chen[2*], Haiying Li[3], Youjia Wu[3], Shouyan Wang[2], Wei Shi[1], Jian Chen[1], Yaohui Ni[4]

1. Department of Neurosurgery, Affiliated Hospital of Nantong University, 20 Xisi Road, Nantong 226001, Jiangsu, China

2. Basic Medical Research Center, Medical School, Nantong University, 19 Qixiu Road, Nantong 226001, Jiangsu, China

3. Department of Pediatrics, Affiliated Hospital of Nantong University, 20 Xisi Road, Nantong 226001, Jiangsu, China

4. Department of Neurology, Affiliated Hospital of Nantong University, 20 Xisi Road, Nantong 226001, Jiangsu, China

Author contributions: J.S and Y.N. designed the research. J.S., X.C., W.S. and J.C. analyzed data. J.S., X.C. and Y.N. wrote the paper.

Data deposition: The microarray data reported in this paper have been deposited in the bioproject at NCBI (https://www.ncbi.nlm.nih.gov/bioproject/?term=PRJNA376061).

The authors declare no conflict of interest.

*These authors contributed equally to this work. Correspondences and requests for materials should be addressed to Y.N. (niyaohui200688@126.com) or X.C. (ylchenxia@ntu.edu.cn)

Supplemental Figure 1. Optimization of siRNA transfection efficiency

Representative images of primary hippocampal cultures transfected with siRNA1 to Itga5 at different concentrations. Left column: phase contrast image. Right column: Cy3 fluorescence.

Supplemental Figure 2. Flow diagram of transcriptome sequencing and bioinformatics analysis pipeline

Supplemental Figures 3-6. Comparisons of co-expression network and identification of pathways

OGD 45 min/R 0 h vs OGD 0 min/R 0 h (Supplemental Fig. 3) OGD 45 min/R 6 h vs OGD 45 min/R 0 h (Supplemental Fig. 4) OGD 45 min/R 12 h (Supplemental Fig. 5) vs OGD 45 min/R 0 h, OGD 45 min/R 18 h vs OGD 45 min/R 0 h (Supplemental Fig. 6). The red and green nodes represent consensus up-regulated or down-regulated terms in the particular pathways, respectively. The yellow nodes represent both up-regulated and down-regulated terms identified in the same particular pathway.

Supplemental Figures 7-10. Co-expression network analysis and identification of key regulatory genes upon oxygen glucose deprivation (OGD) and OGD with reperfusion.

Co-expression network analysis of key regulatory genes for OGD 45 min/R 0 h vs OGD 0 min/R 0 h (Supplemental Fig. 7); OGD 45 min/R 6 h vs OGD 45 min/R 0 h (Supplemental Fig. 8); OGD 45 min/R 12 h vs (Supplemental Fig. 9) and OGD 45 min/R 18 h vs OGD 45 min/R 0 h (Supplemental Fig. 10). The red nodes represented up-regulated genes, and the green nodes represented down-regulated genes.

Supplemental Figure 11. Correlation between qPCR verification and RNA-Seq data

(A-D) Linear regression between results of qPCR and RNA-Seq data for for OGD 45 min/R 0 h vs OGD 0 min/R 0 h (A); OGD 45 min/R 6 h vs OGD 45 min/R 0 h (B); OGD 45 min/R 12 h vs OGD 45 min/R 0 h (C); and OGD 45 min/R 18 h vs OGD 45 min/R 0 h (D).

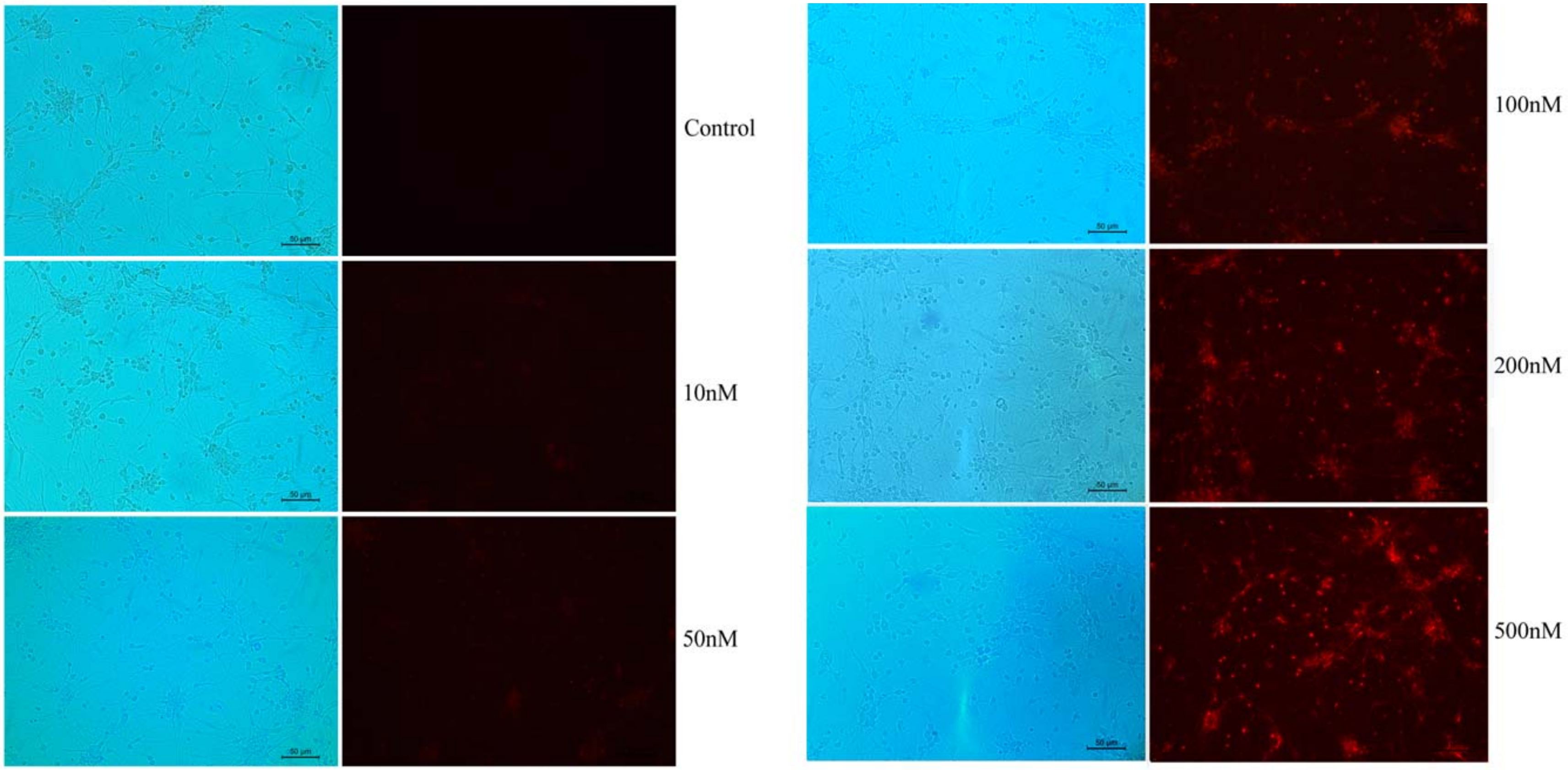

Supplemental Fig. 1

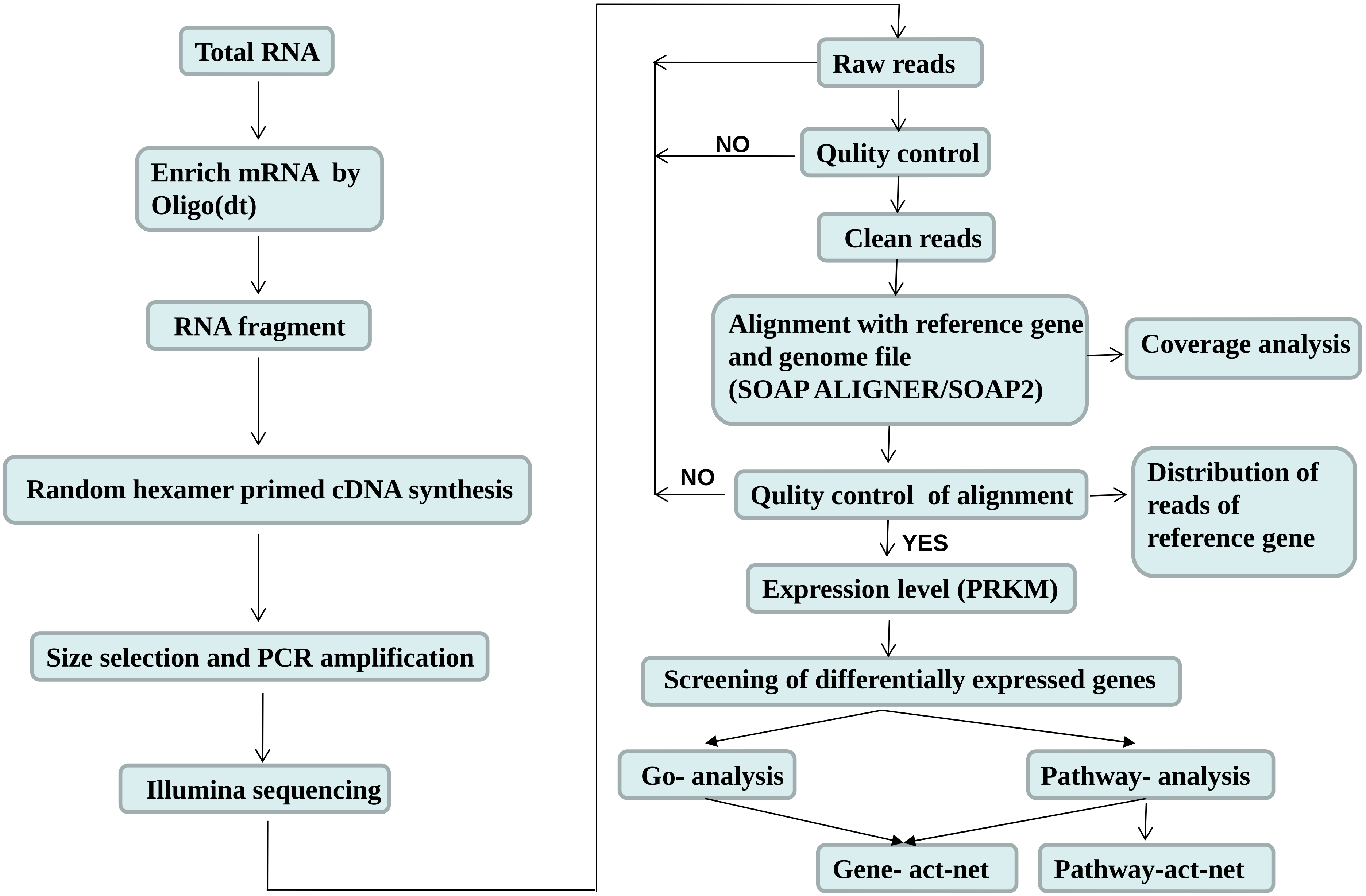

Supplemental Fig. 2

OGD

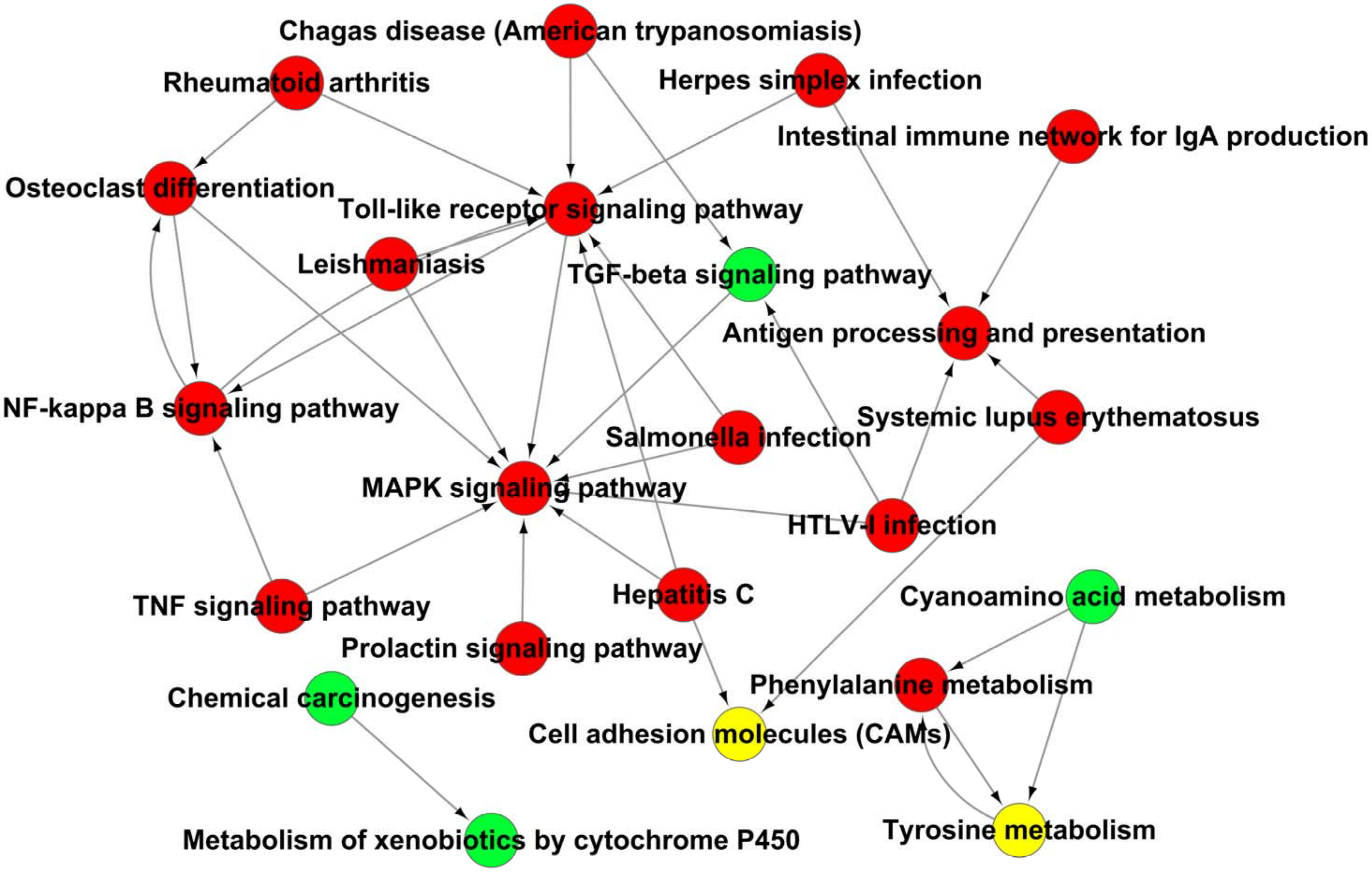

Supplementary Fig. 3

OGD/R 6h

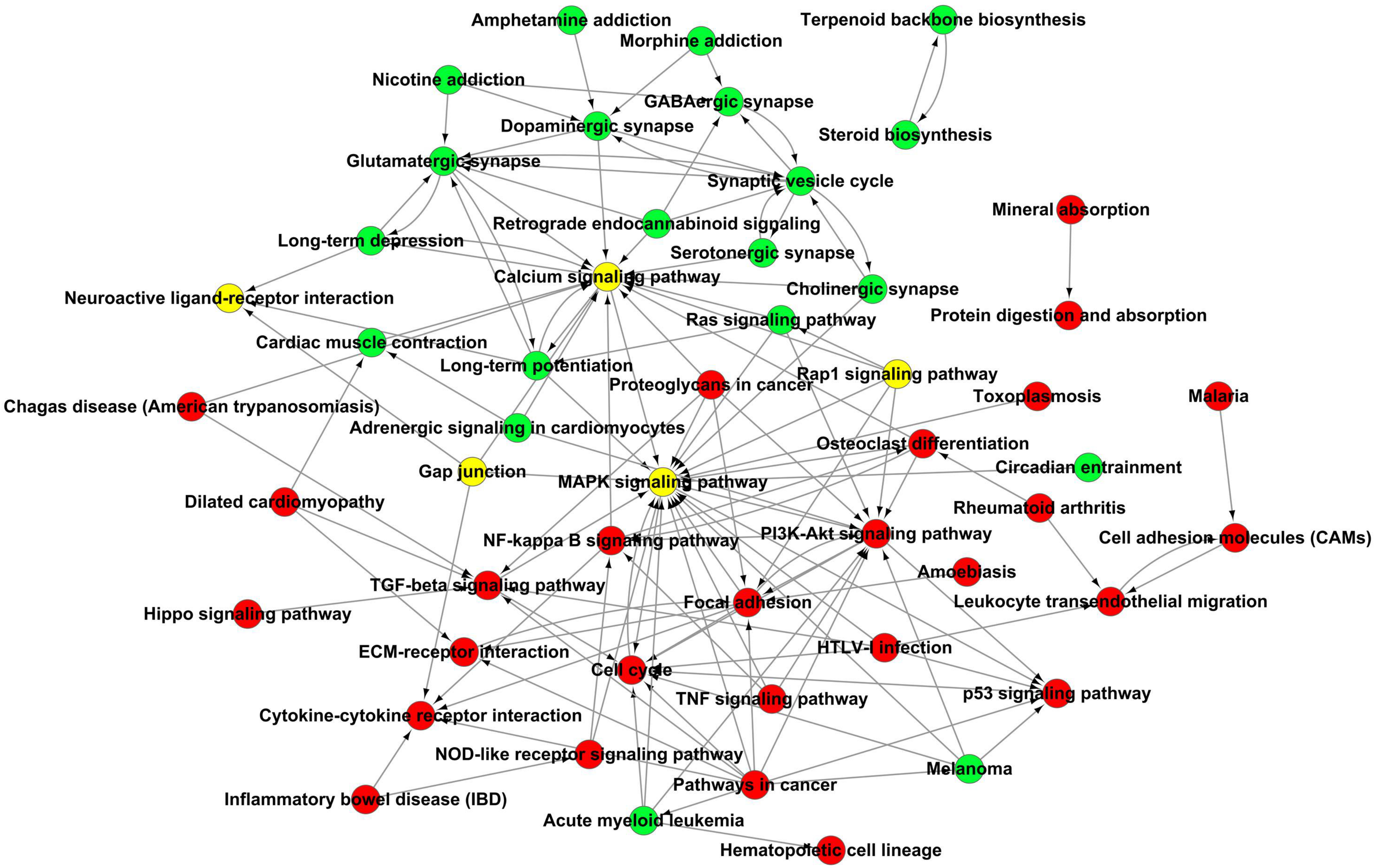

Supplementary Fig. 4

OGD/R 12h

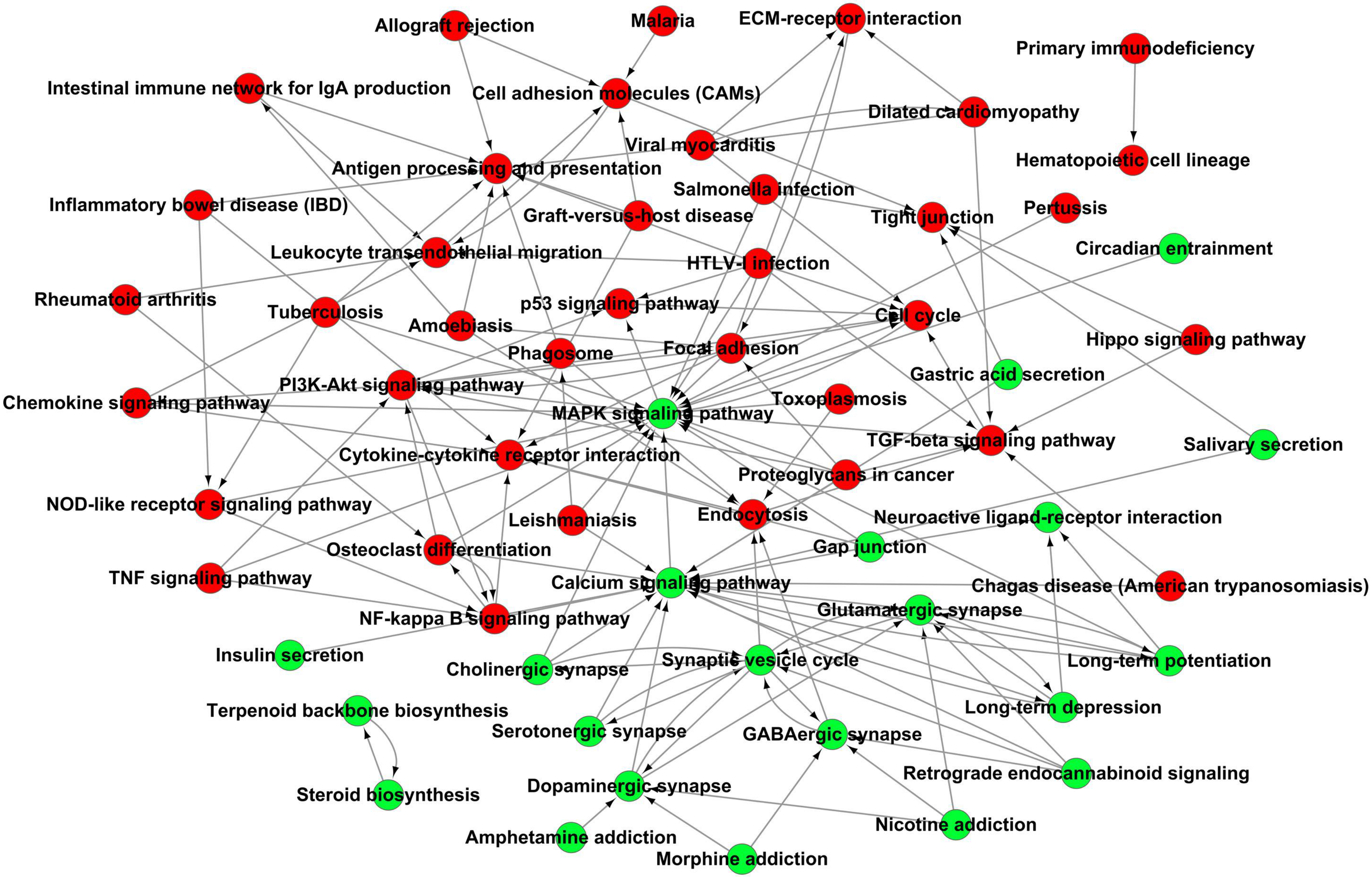

Supplementary Fig. 5

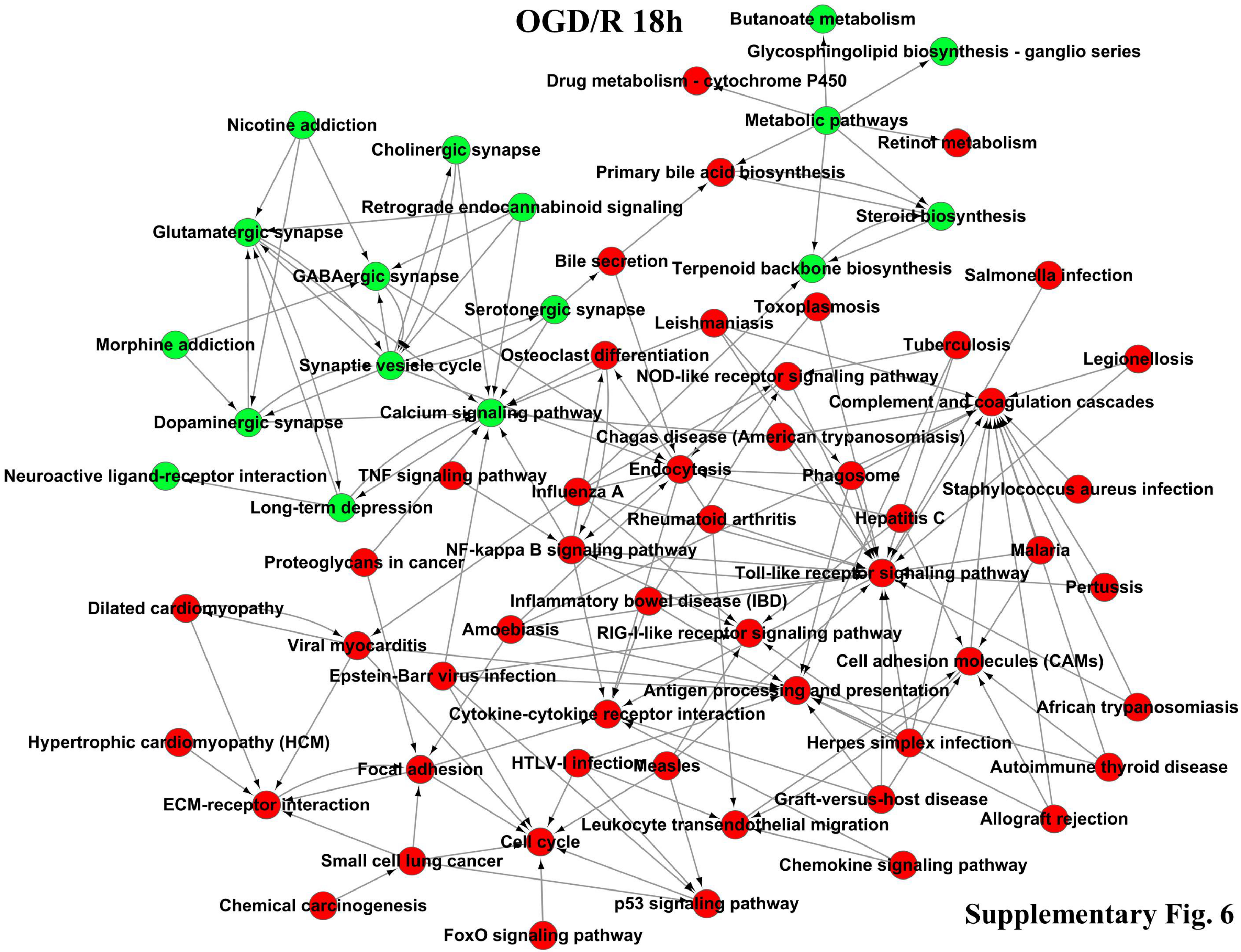

Supplementary Fig. 6

OGD

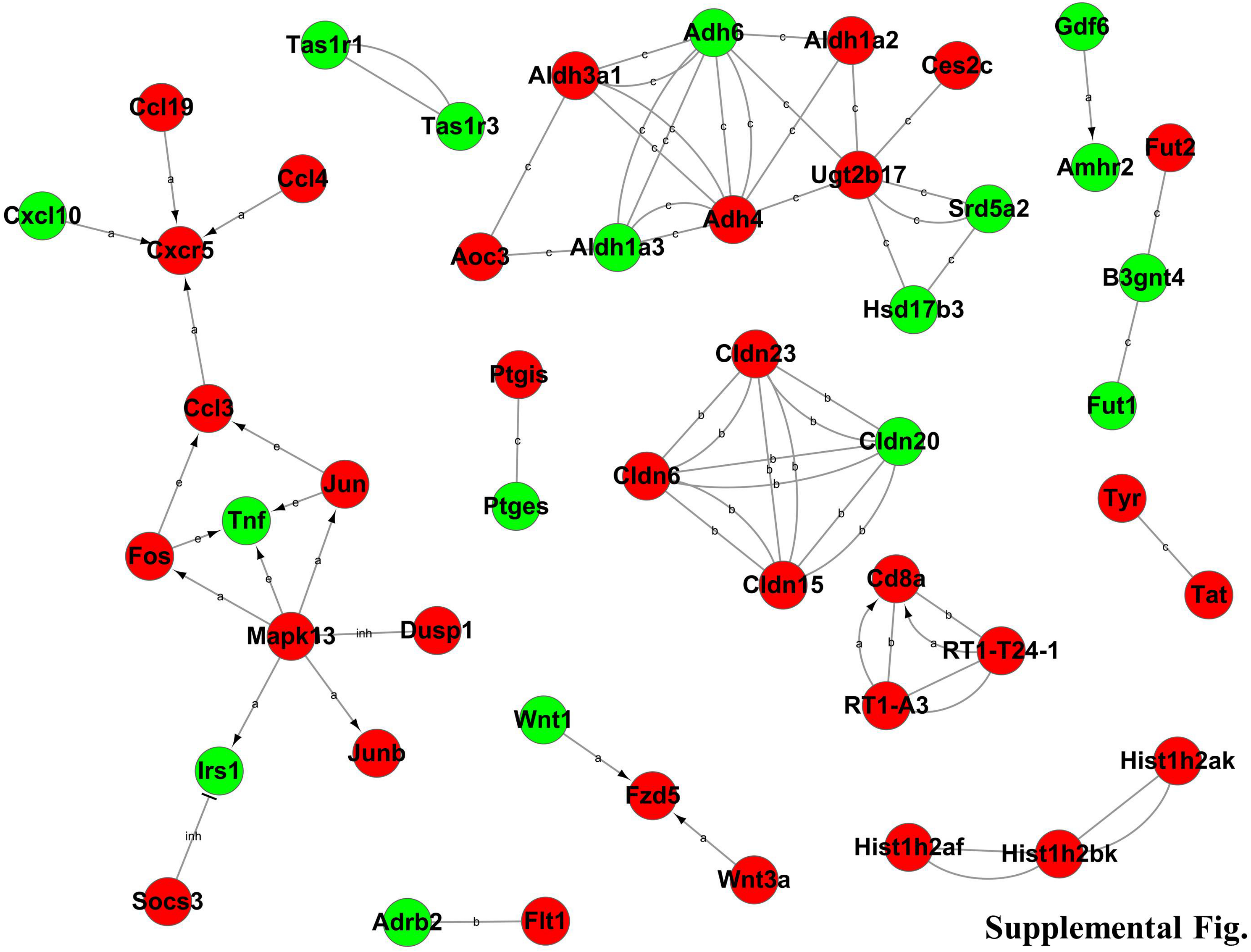

Supplemental Fig. 7

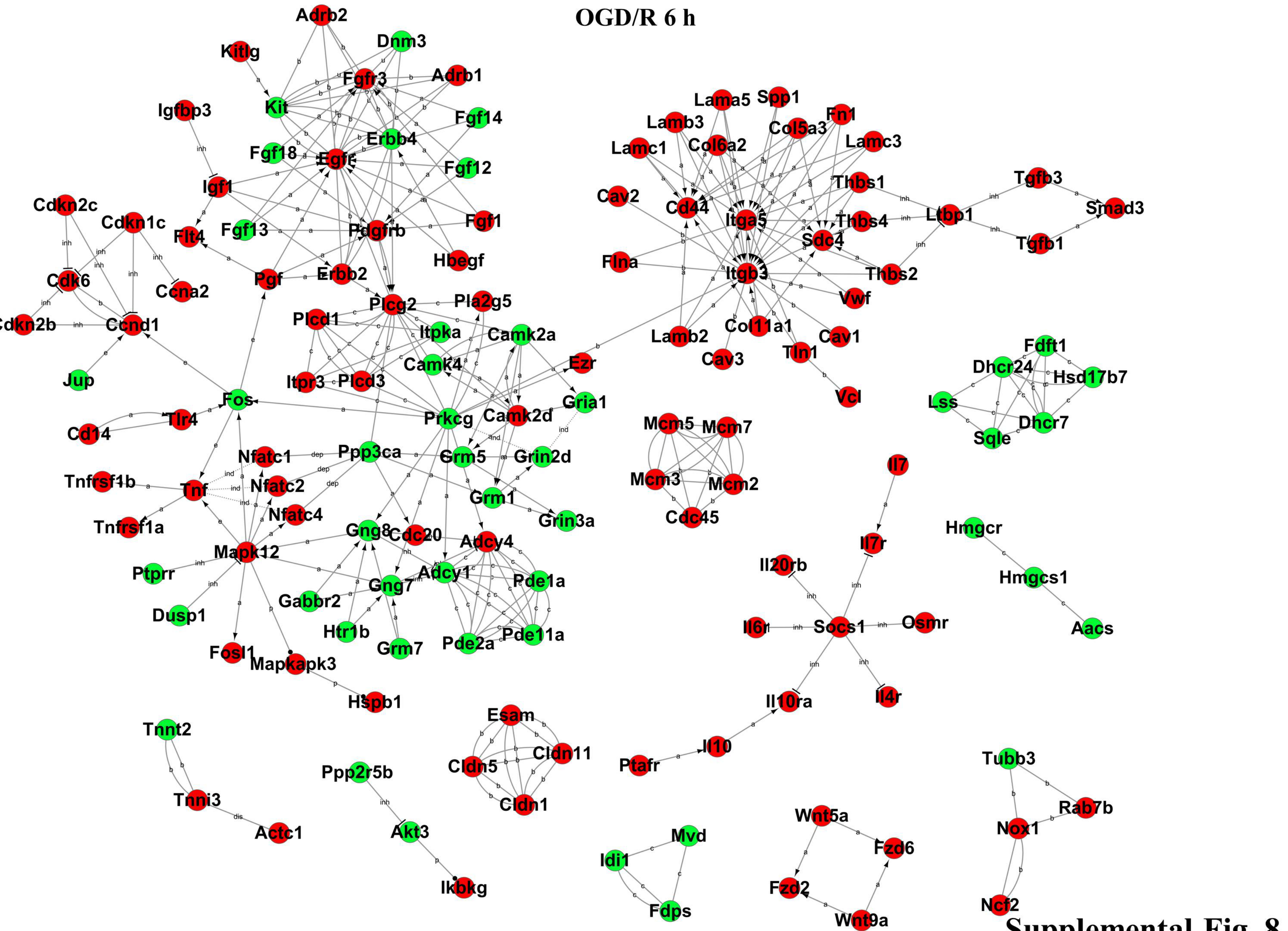

Supplemental Fig. 8

OGD/R 12 h

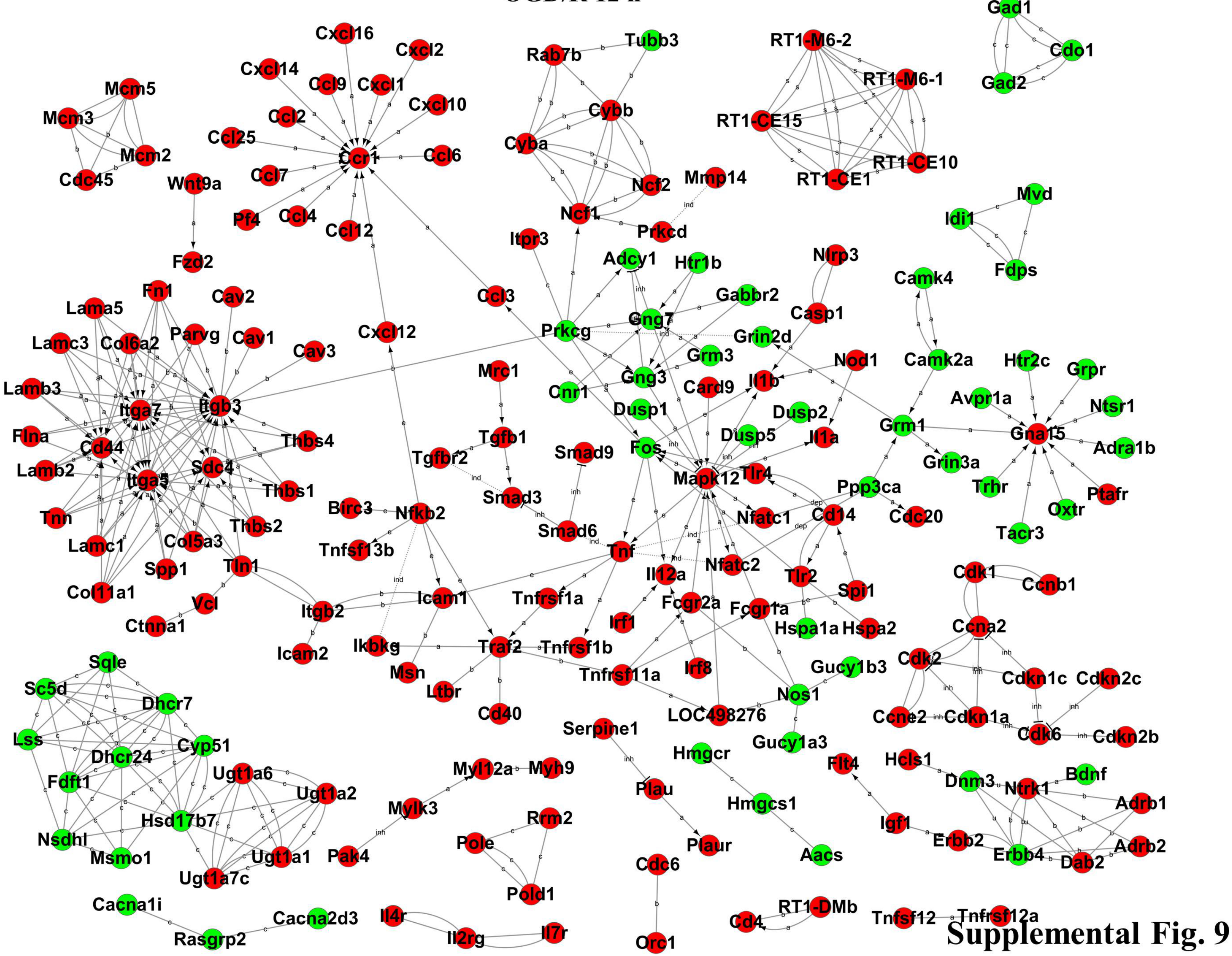

Supplemental Fig. 9

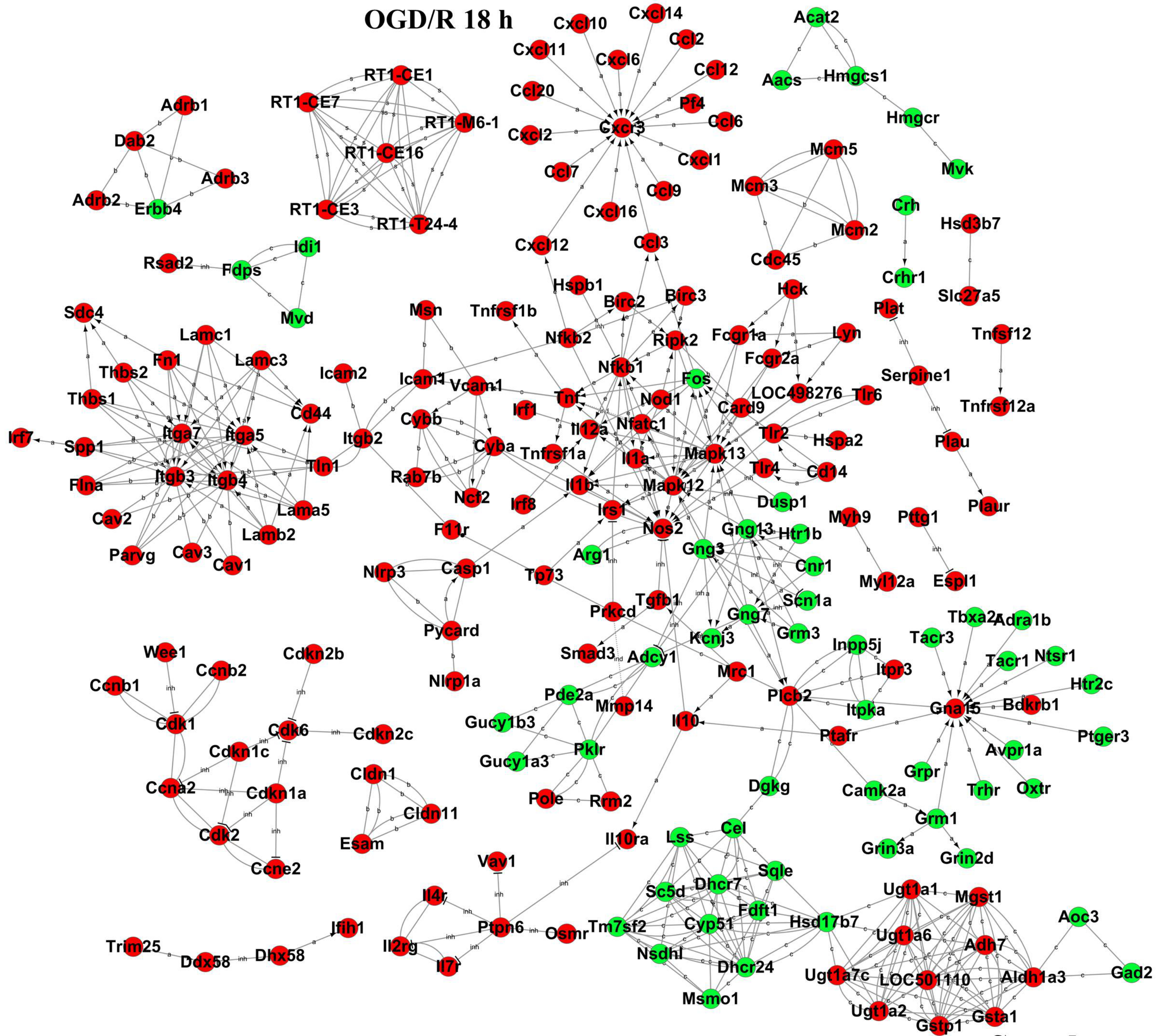

Supplemental Fig. 10

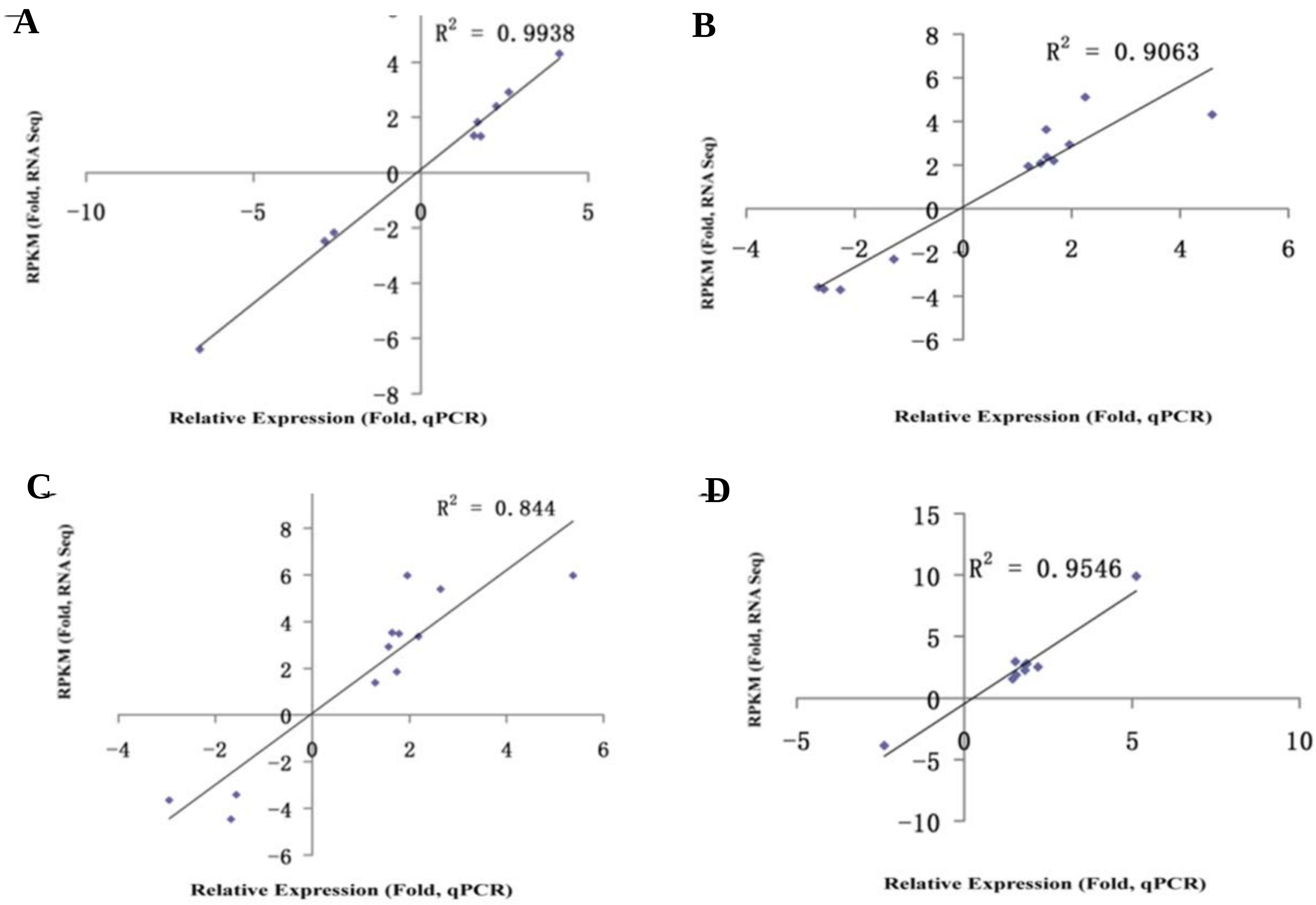

Supplemental Fig. 11

Supplemental Table 2 Primers sequences for qRT-PCR.

NO.	Primers	Sequences（5'-3'）	
		Forward	Reverse
1	Adcy4	ATCAAGGCTCATTGGGCTCC	CCATAGGAAGTCCAGGCGAC
2	Adh6	ACTGTTGGAGCCCTAGCATC	TGAACTGCCTCATTGGCCTC
3	Camk2a	AGATGTGCGACCCTGGAATG	TGAGTGATGCGGATGTAGGC
4	Ccl19	AGCCTTGGCCTCTCAGATTG	TCTTCCGCATCGTTAGCACC
5	Ccl3	TGCTGTTCTTCTCTGCACCAT	CTTGGACCCAGGTCTCTTTGG
6	Ccl4	ATAGGCTCTGACCCTCCCAC	GCACAGATTTGCCTGCCTTTT
7	Cd44	CCCACTTCACAGCCTACTGG	TGCTGTTGTTGTGGGGCTAT
8	Cdk1	GGGAACAGAGAGGGTCCGTT	ATTTCCCGGATTGCCGTACT
9	Cldn15	CTGGGAGTCTCCAACTGCTG	AGCAAGTATGTGGAAGGCCC
10	Erbb4	ACAGCACCCAATCAAGCTCA	GCACTAGGTGTGGGTGATCC
11	Esam	AGGCCACAGCATCAAAACGA	TTCAAAGATCCACGGACGGT
12	Fn1	TTTCCCATTACGCCGTTGGA	TCATCCGCTGGCCATTTTCT
13	Fos	ACTACGAGGCGTCATCCTCC	GTTGGCACTAGAGACGGACA
14	Gdf6	GGTGCCTCACGAGTACATGC	TGAGAGCGTGGACACATCAA
15	Grm1	ACGGCCTGCAAAGAGAATGA	ACAAACAGCGTCACGAGGAT
16	Icam1	TGAGCGACATTGGGGAAGAC	CTACTGAGAGCTGTGTCCGC
17	Il1a	ATCAGCACCTCACAGCTTCC	TCTCCTCCCGATGAGTAGGC
18	Il1b	ACAAGCAACGACAAAATCCC	GACAAACCGCTTTTCCATCT
19	Itga5	CAAGACGCTCCAGTGAGGATT	AGACGTGAGGTCCTGGTTGT
20	Mapk12	CCTTCCAGTCGGAGCTGTTT	CCAGGTCAGTGCCCATGAAT
21	Mapk13	TGCCTTAGAGCGGGAGAAAC	AGCTTCATCCCACTTCGTCG
22	Pla2g5	CTTGGGCTGCCAGCATAAAC	GCAGCCGTAGAAGCCATAGT
23	Spp1	CCGAGGTGATAGCTTGGCTT	CTCTTCATGCGGGAGGTGAG
24	Thbs1	GTGACAAGATGGAGAGCGCA	AGGACGTTGGTTGAACTGGAG
25	Thbs2	ACCAATGCCACCTACCACTG	TGTCCTTCTCATCGCTCACG
26	Tlr4	ATCTGAGCTTCAACCCCCTGA	AGTACCAAGGTTGAGAGCTGG
27	Tnf	CCCAACAAGGAGGAGAAGTT	TTGCTACGACGTGGGCTA

Supplemental Table 3 Selected differentially expressed genes between OGD45min/R0h and OGD0min/R0h cultured neurons

AccID	Description	Degree	log2 Ratio	gene Up-Down	Pathway Term	P-Value	FDR	Enrichment
Cldn15	Claudin-15	6	1.582618974	Up	Cell adhesion molecules (CAMs)	0.003739565	0.08921534	3.81852552
Mapk13	Mitogen-activated protein kinase 13	6	1.688784531	Up	TNF signaling pathway	0.002442273	0.083401036	4.790513834
					Toll-like receptor signaling pathway	0.009937366	0.175787278	4.104022755
					MAPK signaling pathway	0.026403246	0.272998397	2.381724392
Ccl3	C-C motif chemokine 3	3	1.785053369	Up	Toll-like receptor signaling pathway	0.009937366	0.175787278	4.104022755
Ccl4	C-C motif chemokine 4	1	2.260690879	Up	Toll-like receptor signaling pathway	0.009937366	0.175787278	4.104022755
Ccl19	C-C motif chemokine	1	2.639202503	Up	NF-kappa B signaling pathway	0.02603463	0.272998397	3.777466106
Fos	Proto-oncogene c-Fos	3	4.132877251	Up	TNF signaling pathway	0.002442273	0.083401036	4.790513834
					Toll-like receptor signaling pathway	0.009937366	0.175787278	4.104022755
					MAPK signaling pathway	0.026403246	0.272998397	2.381724392
Tnf	Tumor necrosis factor	3	-1.868592138	Down	TGF-beta signaling pathway	0.015445311	0.290252201	6.189989785
Adh6	Alcohol dehydrogenase 6	8	-6.600279954	Down	Tyrosine metabolism	0.029676521	0.290252201	7.984189723
Gdf6	Growth/differentiation factor 6	1	-6.612261242	Down	TGF-beta signaling pathway	0.015445311	0.290252201	6.189989785

Supplemental Table 4 Selected differentially expressed genes between OGD45min/R6h and OGD45min/R0h cultured neurons.

AccID	Description	Degree	log2 Ratio	gene Up-Down	Pathway Term	P-Value	FDR	Enrichment
Thbs2	Protein Thbs2	4	1.208721250	Up	Focal adhesion	1.25643E-07	1.60823E-05	2.77449289
Mapk12	Mapk12 protein	11	1.427740485	Up	Rap1 signaling pathway	0.002784001	0.032324643	1.878227373
					MAPK signaling pathway	0.004474485	0.042424751	1.718373397
					TNF signaling pathway	0.004793678	0.043827916	2.234358797
					NOD-like receptor signaling pathway	0.015001981	0.103797491	2.400190114
Thbs1	Uncharacterized protein	4	1.529887873	Up	Focal adhesion	1.25643E-07	1.60823E-05	2.77449289
					PI3K-Akt signaling pathway	3.2287E-07	2.75516E-05	2.317055037
					ECM-receptor interaction	9.27097E-07	4.93318E-05	3.719031419
					TGF-beta signaling pathway	0.000719331	0.015345734	2.761567053
					Rap1 signaling pathway	0.002784001	0.032324643	1.878227373
					p53 signaling pathway	0.007680619	0.062333575	2.425455273
Adcy4	Adenylate cyclase type 4	11	1.547891516	Up	Rap1 signaling pathway	0.002784001	0.032324643	1.878227373
					Calcium signaling pathway	0.002904167	0.032324643	1.949392986
					Gap junction	0.029860507	0.144775866	1.961006391
Pla2g5	Calcium-dependent phospholipase A2	3	1.671483552	Up	MAPK signaling pathway	0.004474485	0.042424751	1.718373397
					Ras signaling pathway	0.042069125	0.175418163	1.517157208
Cd44	CD44 antigen	10	1.967606107	Up	ECM-receptor interaction	9.27097E-07	4.93318E-05	3.719031419
Itga5	Protein Itga5	18	2.256309291	Up	Focal adhesion	1.25643E-07	1.60823E-05	2.77449289
					PI3K-Akt signaling pathway	3.2287E-07	2.75516E-05	2.317055037
					ECM-receptor interaction	9.27097E-07	4.93318E-05	3.719031419
Tnf	Tumor necrosis factor	7	4.597665486	Up	Cytokine-cytokine receptor interaction	3.98553E-11	1.02029E-08	3.220900282
					NF-kappa B signaling pathway	0.000424993	0.012425797	2.807964349
					TGF-beta signaling pathway	0.000719331	0.015345734	2.761567053
					MAPK signaling pathway	0.004474485	0.042424751	1.718373397
					NOD-like receptor signaling pathway	0.015001981	0.103797491	2.400190114

					Ras signaling pathway	0.015001981	0.042069125	0.175418163
Camk2a	Calcium/calmodulin-dependent protein kinase type II subunit alpha	8	-1.277260327	Down	Calcium signaling pathway	2.36631E-10	2.6266E-08	4.866213542
		8			Dopaminergic synapse	1.37943E-05	0.000382792	3.940982807
					Long-term potentiation	0.00066081	0.008149991	4.163994503
Erbb4	Receptor protein-tyrosine kinase	13	-2.268959603	Down	Calcium signaling pathway	2.36631E-10	2.6266E-08	4.866213542
					MAPK signaling pathway	7.98005E-05	0.001610519	2.669347889
					Ras signaling pathway	0.000726164	0.008484658	2.536095417
					Gap junction	0.000884579	0.009818822	3.642264695
					Rap1 signaling pathway	0.002339552	0.022581765	2.392124935
					PI3K-Akt signaling pathway	0.014925041	0.099282651	1.817062778
Grm1	Metabotropic glutamate receptor 1	5	-2.566792921	Down	Neuroactive ligand-receptor interaction	1.53187E-16	3.40076E-14	5.312682642
					Calcium signaling pathway	2.36631E-10	2.6266E-08	4.866213542
					Glutamatergic synapse	3.28785E-08	1.82476E-06	5.110043005
					Long-term potentiation	0.00066081	0.008149991	4.163994503
					Gap junction	0.000884579	0.009818822	3.642264695
					Long-term depression	0.002526847	0.023373338	3.701328447
Fos	Proto-oncogene c-Fos	6	-2.668593002	Down	Dopaminergic synapse	1.37943E-05	0.000382792	3.940982807
					MAPK signaling pathway	7.98005E-05	0.001610519	2.669347889

Supplemental Table 5 Selected differentially expressed genes between OGD45min/R12h and OGD45min/R0h cultured neurons

AccID	Description	Degree	log2 Ratio	gene Up-Down	PathwayTerm	P-Value	FDR	Enrichment
Icam1	Intercellular adhesion molecule 1	5	1.298448611	Up	NF-kappa B signaling pathway	3.07951E-06	0.000128313	3.445309603
					Cell adhesion molecules (CAMs)	3.81722E-05	0.000636203	2.50931677
					TNF signaling pathway	0.000201274	0.002396115	2.659561129
Fn1	Fibronectin	5	1.569976688	Up	ECM-receptor interaction	4.16222E-06	0.000132053	3.37277677
					Focal adhesion	1.27199E-05	0.000289089	2.332067446
					PI3K-Akt signaling pathway	0.000830533	0.007415474	1.751107686
Itga5	Protein Itga5	19	1.644490656	Up	Focal adhesion	1.27199E-05	0.000289089	2.332067446
Il1b	Interleukin-1 beta	4	1.79216521	Up	Cytokine-cytokine receptor interaction	5.09337E-11	1.27334E-08	3.089543938
					NF-kappa B signaling pathway	3.07951E-06	0.000128313	3.445309603
					NOD-like receptor signaling pathway	7.80545E-06	0.000216818	3.918103448
					TNF signaling pathway	0.000201274	0.002396115	2.659561129
Ccl3	C-C motif chemokine 3	2	1.951336752	Up	Cytokine-cytokine receptor interaction	5.09337E-11	1.27334E-08	3.089543938
					Chemokine signaling pathway	0.002375374	0.016966961	1.957875116
Spp1	Osteopontin	3	2.179380881	Up	ECM-receptor interaction	4.16222E-06	0.000132053	3.37277677
					Focal adhesion	1.27199E-05	0.000289089	2.332067446
					PI3K-Akt signaling pathway	0.000830533	0.007415474	1.751107686
Il1a	Interleukin-1 alpha	2	2.639382492	Up	Cytokine-cytokine receptor interaction	5.09337E-11	1.27334E-08	3.089543938
Tnf	Tumor necrosis factor	8	5.379609955	Up	Cytokine-cytokine receptor interaction	5.09337E-11	1.27334E-08	3.089543938
					NF-kappa B signaling pathway	3.07951E-06	0.000128313	3.445309603
					NOD-like receptor signaling pathway	7.80545E-06	0.000216818	3.918103448
					TGF-beta signaling pathway	0.000113583	0.001494513	2.974041069
Camk2a	Calcium/calmodulin-dependent protein kinase type II subunit alpha	3	-1.566460051	Down	Calcium signaling pathway	4.97807E-09	5.60033E-07	4.30899287
					Cholinergic synapse	6.64634E-05	0.001869282	3.79444519
					Dopaminergic synapse	0.007819491	0.079972067	2.48803292
					Long-term potentiation	0.013301852	0.11084877	2.974024608
Grm1	Metabotropic glutamate receptor 1	5	-1.681575724	Down	Neuroactive ligand-receptor interaction	3.40182E-14	7.6541E-12	4.661746948
					Calcium signaling pathway	4.97807E-09	5.60033E-07	4.30899287

					Glutamatergic synapse	2.40907E-05	0.001355102	3.753992683
					Gap junction	0.001613508	0.02268996	3.344647736
					Long-term depression	0.013301852	0.11084877	2.974024608
					Long-term potentiation	0.013301852	0.11084877	2.974024608
Fos	Proto-oncogene c-Fos	8	-2.952729203	Down	Cholinergic synapse	6.64634E-05	0.001869282	3.79444519
					MAPK signaling pathway	0.000567081	0.009113799	2.344654752
					Dopaminergic synapse	0.007819491	0.079972067	2.48803292

Supplemental Table 6 Selected differentially expressed genes between OGD45min/R18h and OGD45min/R0h cultured neurons

AccID	Description	Degree	log2 Ratio	gene Up-Down	PathwayTerm	P-Value	FDR	Enrichment
Cd44	CD44 antigen	5	1.507596682	Up	ECM-receptor interaction	0.001537207	0.01106789	2.471614935
Cdk1	Cyclin-dependent kinase 1	7	1.521599365	Up	Cell cycle	1.26943E-05	0.00017772	2.872888889
					p53 signaling pathway	0.00017122	0.001598054	3.089518668
Mapk12	Mapk12 protein	20	1.550057886	up	NOD-like receptor signaling pathway	2.02192E-08	2.45663E-06	5.56716218
					TNF signaling pathway	6.1728E-07	2.59258E-05	3.39020979
					Toll-like receptor signaling pathway	0.000392821	0.00341348	2.581675853
					Leukocyte transendothelial migration	0.000628128	0.00510607	2.355529053
					FoxO signaling pathway	0.001472367	0.01091284	2.186066531
					RIG-I-like receptor signaling pathway	0.00646248	0.033928023	2.394074074
Esam	Endothelial cell-selective adhesion molecule	4	1.808660045	Up	Cell adhesion molecules (CAMs)	0.000948451	0.007469053	2.144715188
Spp1	Osteopontin	5	1.813772968	Up	Toll-like receptor signaling pathway	0.000392821	0.00341348	2.581675853
					ECM-receptor interaction	0.001537207	0.01106789	2.471614935
					PI3K-Akt signaling pathway	0.001596964	0.011178747	1.697560044
					Focal adhesion	0.001696697	0.011555883	1.886215595
Icam1	Intercellular adhesion molecule 1	5	1.861984172	Up	TNF signaling pathway	6.1728E-07	2.59258E-05	3.39020979
					NF-kappa B signaling pathway	1.16862E-06	3.68115E-05	3.564378274
					Leukocyte transendothelial migration	0.000628128	0.00510607	2.355529053
					Cell adhesion molecules (CAMs)	0.000948451	0.007469053	2.144715188
Il1a	Interleukin-1 alpha	5	2.198418136	Up	Cytokine-cytokine receptor interaction	1.73319E-10	4.36765E-08	3.007444169
Tnf	Tumor necrosis factor	9	5.119447582	Up	Cytokine-cytokine receptor interaction	1.73319E-10	4.36765E-08	3.007444169
					NF-kappa B signaling pathway	1.16862E-06	3.68115E-05	3.564378274
					RIG-I-like receptor signaling pathway	0.00646248	0.033928023	2.394074074
					TGF-beta signaling pathway	0.010018741	0.048552358	2.172668779
Fos	Proto-oncogene c-Fos	8	-2.395136975	Down	Cholinergic synapse	0.000508371	0.009659048	3.631312306

Dopaminergic synapse	0.001963335	0.024137468	3.03044768
Estrogen signaling pathway	0.007481381	0.071073122	3.033175355
MAPK signaling pathway	0.016543222	0.137109791	1.947144349
Oxytocin signaling pathway	0.036372062	0.245218092	2.101491157
